# Population analysis and the effects of Gaussian basis set quality and quantum mechanical approach: main group through heavy element species

**DOI:** 10.3389/fchem.2023.1152500

**Published:** 2023-04-19

**Authors:** Sasha C. North, Kameron R. Jorgensen, Jason Pricetolstoy, Angela K. Wilson

**Affiliations:** ^1^ Department of Chemistry, Michigan State University, East Lansing, MI, United States; ^2^ Department of Biology and Chemistry, Texas A&M International University, Laredo, TX, United States

**Keywords:** electrostatic potential (ESP) derived charges, volume-based methods, atomic charge, population analysis, orbital-based methods

## Abstract

Atomic charge and its distribution across molecules provide important insight into chemical behavior. Though there are many studies on various routes for the determination of atomic charge, there are few studies that examine the broader impact of basis set and quantum method used over many types of population analysis methods across the periodic table. Largely, such a study of population analysis has focused on main-group species. In this work, atomic charges were calculated using several population analysis methods including orbital-based methods (Mulliken, Löwdin, and Natural Population Analysis), volume-based methods (Atoms-in-Molecules (AIM) and Hirshfeld), and potential derived charges (CHELP, CHELPG, and Merz-Kollman). The impact of basis set and quantum mechanical method choices upon population analysis has been considered. The basis sets utilized include Pople (6-21G**, 6-31G**, 6-311G**) and Dunning (cc-pV*n*Z, aug-cc-pV*n*Z; *n* = D, T, Q, 5) basis sets for main group molecules. For the transition metal and heavy element species examined, relativistic forms of the correlation consistent basis sets were used. This is the first time the cc-pV*n*Z-DK3 and cc-pwCV*n*Z-DK3 basis sets have been examined with respect to their behavior across all levels of basis sets for atomic charges for an actinide. The quantum methods chosen include two density functional (PBE0 and B3LYP), Hartree-Fock, and second-order Møller-Plesset perturbation theory (MP2) approaches.

## 1 Introduction

Atomic charge is important in the rationalization and elucidation of chemical and structural characteristics of molecules. How this charge is distributed across an atom influences the bonding between (intermolecular) and within (intramolecular) molecules. Insight about the electronic density, and thus, the distribution of the charge across an atom, can be quantified using population analysis approaches.

Population analysis has proven useful for decades to help provide insight about a broad range of chemistry. To provide a number of examples, in recent work, population analysis has been used to elucidate resins that are more effective for cadmium removal (Zhang et al., 2022) to help understand the dynamics of methanol’s main reaction channels (Catone et al., 2021) and to provide insight about the interaction of chloroquine with C_60_ (Novir and Aram, 2020). For a study on endocannabinoid receptors, a therapeutic target for physiological pain treatment and mood regulation, population analysis was used to help indicate the most probable sites to undergo a nucleophilic attack (Rangel-Galván et al., 2022). Electrostatic partial charges, obtained from population analysis, have been used in the parameterization and the development of force fields, essential to molecular dynamics (MD) simulations (Bai et al., 2022; da Silva et al., 2022; Kognole et al., 2022; Uene et al., 2022).

Widely used population analysis schemes include: A) those based on wavefunction partitioning and orbital based schemes [Mulliken (MPA) (Mulliken, 1955), Löwdin (LPA) (Löwdin, 1970), and Natural Population Analysis (NPA) (Reed et al., 1985)]; B) volume-based charge assignment methods [Atoms-in-Molecules (AIM) (Bader, 1990) and Hirshfeld population analysis (Hirshfeld, 1977)]; and, C) electrostatic potential (ESP) approaches [CHELP (Chirlian et al., 1987), CHELPG (Breneman and Wiberg, 1990), and Merz-Kollman (MK) population analysis (Singh and Kollman, 1984; Besler et al., 1990)]. There is a very rich history of population analysis schemes, far too rich to address here. However, a brief overview of these methods and illustrations of modifications that have been made for some of the methods are provided here.

### 1.1 Wavefunction partitioning and orbital based schemes

For the population methods that are based on wavefunction partitioning and are orbital based (Type A), Mulliken population analysis (Mulliken, 1955) uses orbital partitioning schemes accounting for atomic orbital overlap and overlap population. The Mulliken charge assigned to atom k is calculated using the difference between the atomic number of atom k (Z_k_) and the sum over basis functions centered on atom k plus the overlap contribution from basis set functions centered at other atoms (Eq. 1).

$$Q_{k}=Z_{k}-\left(\underset{i\in k}{\sum }P_{i,i}+\underset{i\in k}{\sum }\underset{j\neq i}{\sum }{S_{ij}P}_{ij}\right)$$ (1)

Errors arise due to the Mulliken method evenly dividing the overlap population between two atoms without considering atom type or electronegativity (Though there have been suggested modifications to the original Mulliken method such as work by Noell for application to transition metal complexes) (Noell, 1982). In a number of studies, Mulliken charges have been shown to have basis set dependence which can yield inconsistent and poor charge values, depending upon basis set chosen (Mulliken, 1971; Cramer, 2002; Jensen, 2007). For example, in the water molecule, the partial charge on the oxygen atom varied by 0.5263 *e* when using a small basis set (aug-cc-pVDZ) *versus* using a large basis set (aug-cc-pV5Z) (Martin and Zipse, 2005). However, when using Atoms-in-Molecules (AIM), a topological method (described below), the difference in charge assignment between the small and large basis sets was only 0.0078 *e* for oxygen (Martin and Zipse, 2005). Despite the possible basis set dependencies, Mulliken charges continue to be used due to the simplicity of this charge assignment scheme.

Other wavefunction based methods have sought to improve upon Mulliken based charges including Löwdin population analysis (LPA) (Löwdin, 1970), and Natural Population Analysis (NPA) (Reed et al., 1985). LPA uses the Löwdin symmetrically orthogonalized atomic orbitals to assign the electron density maintaining the essential features of Mulliken analysis to reduce basis set dependencies (Mayer, 2004). NPA addresses the basis set dependence of Mulliken by utilizing the Natural Bond Order approach (Foster and Weinhold, 1980; Reed and Weinhold, 1983; Reed and Weinhold, 1985), a bond analysis technique. NPA is based on the construction of a set of “natural atomic orbitals” (NAOs) for a given molecule using an arbitrary atomic orbital basis set. The “natural population analysis” simply represents the occupancies (diagonal one-particle density matrix elements) of these NAOs in the system of interest and enables greater numerical stability and provides a better description of the electronic distribution in compounds with high ionic character than the Mulliken approach.

### 1.2 Volume based charge assignment methods

(Type B) include Atoms-in-Molecules (AIM) and Hirshfeld population analysis. These methods assign charge based on the volumes occupied by each atom. These calculations are more demanding than Mulliken analysis. For example, the AIM (Bader, 1990) approach divides molecular charge density into atomic contributions based on topology. This requires knowledge of the gradient vector field of the charge densities, and the division of the three-dimensional space of the molecule into atomic volumes containing exactly one nucleus, which acts as a local attractor of the electron density. Hirshfeld population analysis (Hirshfeld, 1977; Ritchie, 1985; Ritchie and Bachrach, 1987) gives the total density of a molecule as a sum of well-defined contributions from its constituent atoms, and thus simply divides the molecular density at each atomic “point” in the molecule in proportion to their respective contributions to the molecular density. Hirshfeld charges have been shown to decrease basis set dependence, however, Hirshfeld charges have been shown to be smaller in absolute value than expected for atomic charges. Among the first to note this difference were Davidson and Chakravorty (1992) and Bultinck et al. (2007) For example, in work by Bultinck, the Hirshfeld charges on the atoms in the (LiNH_3_)^+^ ion were found to be 0.751 for Li, −0.203 for N, and 0.151 for H when ROHF/6-311G** was used, which are smaller than anticipated.

### 1.3 Electrostatic potential (ESP) approaches

(Type C) rely on the partitioning of electrostatic potentials. These types of methods result in less basis set dependence compared to orbital based methods and are less computationally expensive as compared to topology based methods (Chirlian et al., 1987). The partitioning is accomplished through fitting points across the molecular coordinate system to reproduce the electrostatic potential. Common ESP methods including CHELP (Chirlian et al., 1987), CHELPG (Breneman and Wiberg, 1990), and Merz-Kollman (MK) (Singh and Kollman, 1984; Besler et al., 1990) differing in their grid points choice and fitting procedure method for the potential. CHELP assigns points on spherical shells around each atom (14 points per shell) at 2.5, 3.5, 4.5, 5.5, and 6.5 Å from each atom excluding points in the van der Waals radii (the radius of an imaginary hard sphere representing the distance of closest approach for another atom) for any atom. CHELPG assigns points on an evenly spaced cubic grid including a much denser packing of points (1 point per 0.3 Å) than CHELP and MK methods, sampling points between 0 and 2.8 Å around each atom, including the van der Waals radii. MK uses nested Connolly surface algorithms, assigning one to five points every 1 Å, including points by scaling atomic radii to multiples of (1.4, 1.6, 1.8 and 2.0) the van der Waals radii, and discarding all points inside the van der Waals volume. Weaknesses of charges determined using ESP methods include that they are not easily transferable between common (molecular) functional groups in related molecules, they have often been conformationally dependent, and large charges can result. The restricted electrostatic potential (RESP) model (Bayly et al., 1993) has been developed for molecule charge assignment to address some of these challenges. Numerous studies have been made using ESP charge assignment models to calculate charge of a single molecule, or of several molecules at a specified Hamiltonian and basis set (Sigfridsson and Ryde, 1998).

### 1.4 Advances in population analysis methods: Examples

In considering population analysis schemes, many studies have been done to extend or more thoroughly understand the methods. For example, in a critical analysis of Hirshfield population analysis by Bultinck et al. (2007), Bultinck identified a number of weaknesses with the analysis approach, pointing out the unphysical nature of some of the predictions, and the methodology selected for computational convenience. As a result, an extension to the Hirshfeld method was suggested by Bultinck, utilizing a weighted sums of charge atomic densities in an iterative procedure until self-consistent charges are determined. In 2006, Bruhn et al. (2006) used atomic orbital-based population analyses, examining the way these methods are affected by rigid rotation of the molecule. They demonstrated that the Mulliken and pre-orthogonalized Löwdin population analyses are invariant to a general rotation transformation, while the standard non-pre-orthogonalized implementation of the Löwdin formalism may not be invariant to such rotations. In a different study, Hirshfeld and Mulliken population analysis methods were examined to determine if the charge assignment within the methods was consistent with chemical intuition (i.e., consistent with commonly used chemical concepts such as electronegativity). The study suggested improvements for the Hirshfeld method charge partitioning technique such as increasing the magnitude of the atomic charges (Saha et al., 2009). In 2020, a study was done by Cho et al. (2020) that examined the use of a broad range of population analysis methods on the GMTKN55 set of nearly 2,500 main-group molecules using the PBE0 density functional.

In addition to the numerous studies of population methods, there have been many reviews of population analysis schemes. These include works by Wiberg and Rablen, who compared charges obtained using Mulliken population analysis, Natural Population Analysis (NPA), Bader’s Atoms-in-Molecules (AIM), CHELPG, and using atomic polar tensors (GAPT), and applied to hydrocarbons (Wiberg and Rablen, 1993). The same authors revisited population analysis schemes 25 years later, when they compared charges obtained using Mulliken, minimal basis set (MBS), NPA, Mertz-Kollman (MK), CHELPG, Hirshfeld, and charge model 5 (CM5) methods, applied to hydrogen charges and hydrogen bonding (Wiberg and Rablen, 2018). Comparison to experimental results from high-resolution spectroscopic studies of deuterated methanes and known energies for H···O hydrogen bonds demonstrated that the Hirshfeld charges were the most reliable as compared to experiment. Bachrach (1994) gave a lengthy review in 1994 on commonly used population methods in “Population Analysis and Electron Densities from Quantum Mechanics.” Heidar-Zadeh et al. (2018) reviewed population analysis schemes using information theory, and recently Davidson and Clark (Davidson and Clark, 2022) discussed the historical context that has influenced common conceptions about chemical bonding and reactivity (such as charge), as well as relevant technical considerations of population analyses (primarily from the Schrödinger perspective). For example, the isolated atom does not appear in the Schrödinger equation of a molecule, and thus atomic properties (such as atomic populations) must be obtained from post-processing partitioning of the wavefunction. Therefore, Davidson and Clark discuss algebraic considerations of this partitioning, such as orbital and spatial decomposition schemes of the density matrix.

While there are many studies utilizing population analysis techniques, useful illustrations are needed to demonstrate the role of basis set and quantum mechanical method choice on the assignment of atomic charge across many population analysis schemes. For example, though Cho et al. (2020) examined a wide variety of population analysis schemes and a large, diverse set of molecules, only DFT with the PBE0 functional was considered. Similarly, Wiberg and Rablen considered many classes of population schemes in their works (Wiberg and Rablen, 1993; Wiberg and Rablen, 2018) but focused on the performance of these schemes for hydrogen charges and hydrogen bonding. Another study considered both the basis set and quantum method dependance of the AIM method, using HF and DFT with the B3LYP functional, and used both Dunning and Pople basis sets, but did not consider additional population methods (Jabłoński and Palusiak, 2010). Though these are only a few examples of the many prior studies, they do illustrate the typical focused nature of much of the prior studies.

In the present study, the assignment of charge using several different basis sets, quantum mechanical methods, and molecule type, as well as the effect of molecular polarity on charges, will be examined in this work. Although much development has been done on some of these population analysis methods, such as the iterative Hirshfeld (Bultinck et al., 2007) and pre-orthogonalized Löwdin (Bruhn et al., 2006), this work considers population analysis schemes in their original descriptions, examining their performance with a variety of basis sets and quantum mechanical methods, as these are the methods that are most commonly used by the chemistry community. The current work includes main group, transition metal, and heavy element species, considering the impact of basis set choices, cc-pV*n*Z-DK3 and cc-pwCV*n*Z-DK3 (Peterson, 2015; Feng and Peterson, 2017), for heavier element species.

## 2 Computational methods

Atomic charges for HF, LiF, MgO, NaCl, SO_2_, CO_2_, H_2_O, BeCl_2_, MgCl_2_, NH_3_, BF_3_, CH_4_, VO, and LrF have been determined. This set of molecules represents a variety of bonding, oxidation states, and structures. Bond electronegativity differences range from 0.45 (C—H) to 3.00 (Li—F) for the main group molecules. Four different *ab initio* and density functional approaches have been used including the B3LYP and PBE0 (Adamo and Barone, 1999) functionals, Hartree-Fock (HF), and second-order Møller–Plesset perturbation theory (MP2).

For main group molecules, the Dunning correlation consistent (cc-pV*n*Z, aug-cc-pV*n*Z, *n* = D,T,Q,5) (Dunning, 1989; Kendall et al., 1992; Prascher et al., 2011) and Pople (6-21G**, 6-31G**, 6-311G**) (Hehre et al., 1972; Hariharan and Pople, 1973; Dill and Pople, 1975; Binkley and Pople, 1977; McLean and Chandler, 1980; Raghavachari et al., 1980; Francl et al., 1982; Gordon et al., 1982) basis sets were used. The *tight-d* basis sets, cc-pV (*n*+*d*)Z, and aug-cc-pV (*n*+*d*)Z (*n* = D,T,Q,5) (Dunning et al., 2001), were used for second-row atoms. Experimental geometries from the NIST Computational Chemistry Comparison and Benchmark Database were used for all calculations (Johnson, 2015). The correlation consistent (cc-pV*n*Z, aug-cc-pV*n*Z, *n* = D,T,Q,5) basis sets (Balabanov and Peterson, 2005; Balabanov and Peterson, 2006), were used for vanadium oxide. For the lawrencium fluoride calculations, cc-pVnZ-DK3 and cc-pwCV*n*Z-DK3 basis sets were used for lawrencium, and the cc-pVnZ-DK basis sets were used for fluorine (*n* = D,T,Q for each atom) (Peterson, 2015; Feng and Peterson, 2017). For LrF, wavefunction based and volume-based population analysis were the focus of the calculations.

Mulliken, Löwdin, NPA, Hirshfeld, AIM, CHELP, CHELPG, and MK population analysis have been done, using Gaussian 16 (Frisch et al., 2016). CHELP initial radii values that were not predefined in Gaussian were set as the covalent radii for beryllium and boron (1.06 and 0.83 Å, respectively) and ionic radii for lithium, sodium, magnesium, and vanadium (0.90, 1.16, 0.86, and 0.93 Å, respectively) (Teatum et al., 1960; Allen et al., 1987). The atom charges for the center atoms in the polyatomic molecules investigated are given (e.g., C in CH_4_, O in H_2_O) in the present work. For diatomics, cation charges were considered for MgO, NaCl, VO and LrF, and the anion charges were considered for HF and LiF. Orbital-based (Mulliken, Löwdin, NPA) and volume-based (Hirshfeld and AIM) methods were utilized for LrF.

## 3 Results and discussion

### 3.1 Main group molecules

#### 3.1.1 Polar bonds

**FIGURE 1:**
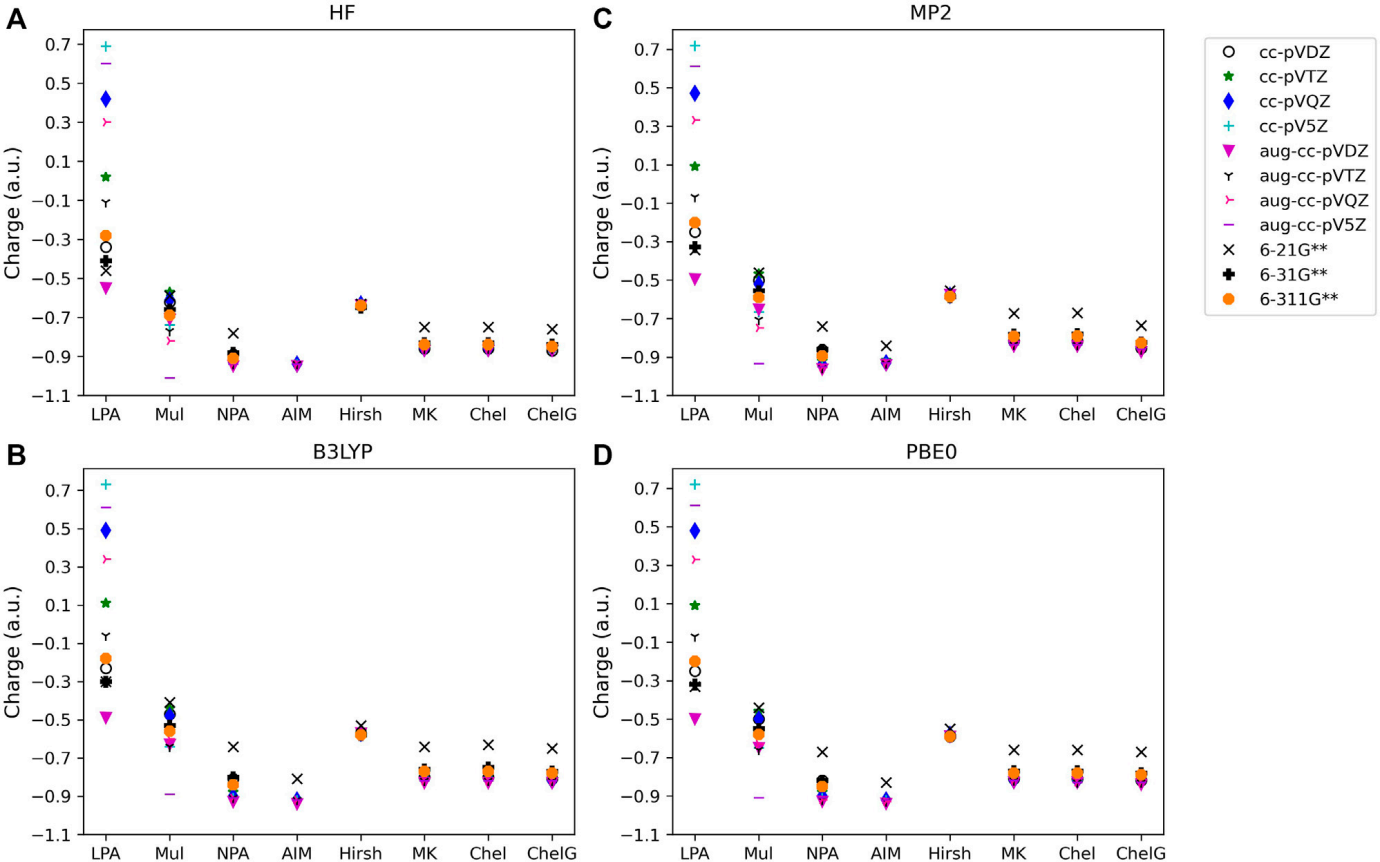
Atomic charge determined for noted basis set, analysis method, and quantum mechanical method: **(A)** HF, **(B)** B3LYP, **(C)** MP2, and **(D)** PBE0 for one of the diatomic compounds investigated, LiF. Charges for the remaining three diatomic molecules studied are included in the SI.

**FIGURE 2:**
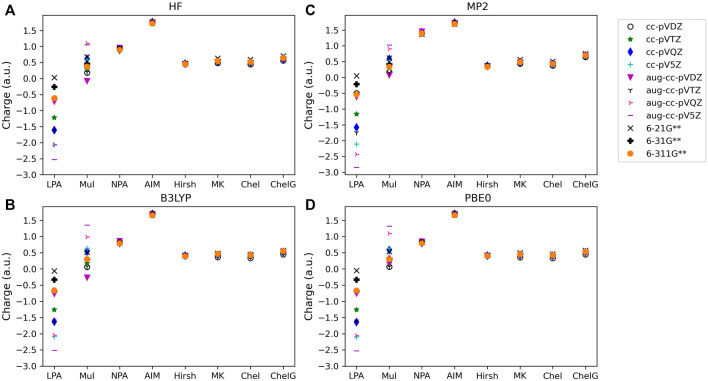
Atomic charge determined for noted basis set, analysis method and quantum mechanical method: **(A)** HF, **(B)** B3LYP, **(C)** MP2, and **(D)** PBE0 for one of the triatomic compounds investigated, BeCl2. Charges for the remaining four triatomic compounds studied are included in the SI.

**FIGURE 3:**
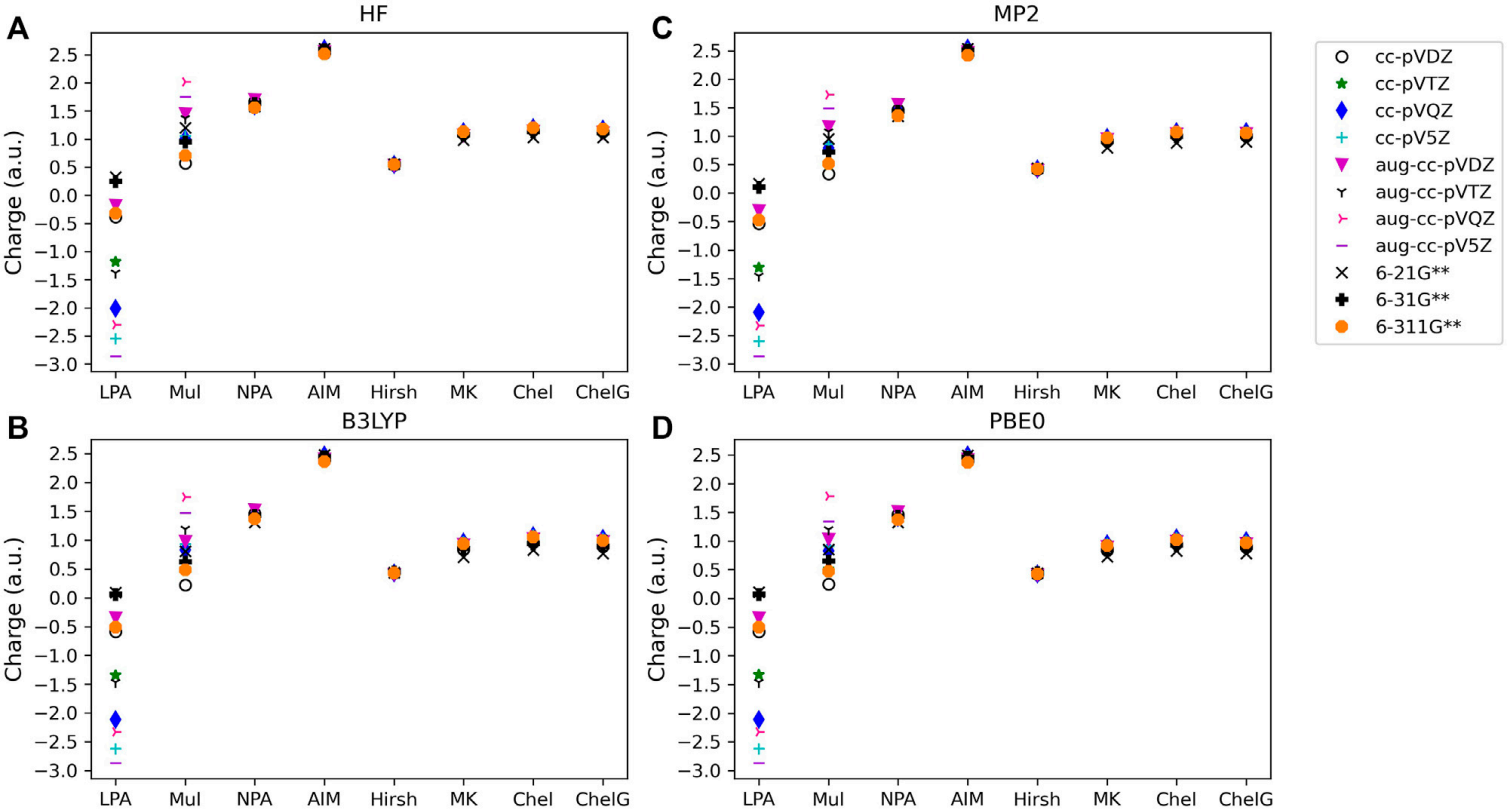
Atomic charge determined for noted basis set, analysis method and quantum mechanical method: **(A)** HF, **(B)** B3LYP, **(C)** MP2, and **(D)** PBE0 for one of the triatomic compounds investigated, BF_3_. Charges for the two remaining polyatomic compounds are included in the SI.

The atomic charge (q_c_) determined for each type of molecule investigated, along with method, basis set, and analysis used are provided in Figures 1–3. Figure 1 shows the charges for LiF; Figure 2 provides charges for BeCl_2_,; Figure 3 gives the charges for BF_3_. Charges for the other main group molecules are included in the tables in the Supporting Material (SI).

To consider the assigned charges for molecules with quite different bond polarities, Tables 1 and 2 shows the fluorine charges for the HF and much more polar LiF, molecules. The difference in electronegativity between hydrogen and fluorine is 1.88, and between lithium and fluorine is 3.00. The maximum charge difference between the fluorine in HF and in LiF is 0.82 *e*, as given by Löwdin population analysis using PBE0/aug-cc-pVQZ. On average across all methods there is a fluorine charge difference in HF compared to LiF of 0.34 *e*. The most consistent (least basis set dependent) of the methods is the Hirshfeld method which, on average, results in a fluorine charge difference of 0.37 *e* between HF and LiF. The orbital-based methods are basis set dependent and slightly more so for the polar molecules. The difference between the charges obtained using the various cc-pVnZ basis sets for LiF with PBE0 is 0.97 *e*, while for the HF molecule, the maximum difference between charges obtained using PBE0 and the cc-pV*n*Z sets is 0.91 *e*. Similarly, the maximum difference between charges obtained using HF/cc-pVnZ is 1.03 *e* for LiF, while it is 0.92 *e* for HF. Hirshfeld and electrostatic potential based methods show much less dependence on basis set as compared to orbital based methods.

**TABLE 1:** Comparison of charges obtained for the most polar (LiF) and least polar (HF) diatomic molecules, using different quantum methods, population analysis schemes, and basis sets.

		Fluorine charge in LiF (ΔEN = 3.00)								Fluorine charge in HF (ΔEN = 1.88)							
		Q_LPA_	Q_MPA_	Q_NPA_	Q_AIM_	Q_HPA_	Q_MK_	Q_CHELP_	Q_CHELPG_	Q_LPA_	Q_MPA_	Q_NPA_	Q_AIM_	Q_HPA_	Q_MK_	Q_CHELP_	Q_CHELPG_
B3LYP	cc-pVDZ	−0.23	−0.47	−0.81	*	−0.57	−0.80	−0.80	−0.81	−0.06	−0.21	−0.52	−0.72	−0.23	−0.41	−0.40	−0.41
	cc-pVTZ	0.11	−0.44	−0.88	−0.92	−0.57	−0.81	−0.81	−0.81	0.25	−0.32	−0.54	−0.72	−0.22	−0.42	−0.40	−0.42
	cc-pVQZ	0.49	−0.48	−0.90	−0.92	−0.57	−0.81	−0.81	−0.82	0.62	−0.34	−0.55	−0.72	−0.21	−0.42	−0.40	−0.42
	cc-pV5Z	0.73	−0.64	−0.91	−0.92	−0.57	−0.82	−0.82	−0.82	0.85	−0.37	−0.54	−0.73	−0.21	−0.42	−0.40	−0.42
	aug-cc-pVDZ	−0.49	−0.63	−0.93	−0.94	−0.57	−0.83	−0.83	−0.83	0.09	−0.25	−0.56	−0.71	−0.21	−0.41	−0.39	−0.41
	aug-cc-pVTZ	−0.06	−0.64	−0.91	−0.92	−0.57	−0.82	−0.82	−0.82	0.66	−0.33	−0.55	−0.73	−0.21	−0.41	−0.40	−0.41
	aug-cc-pVQZ	0.34	−0.60	−0.91	−0.92	−0.57	−0.82	−0.82	−0.82	1.13	−0.43	−0.55	−0.73	−0.21	−0.41	−0.40	−0.41
	aug-cc-pV5Z	0.61	−0.89	−0.91	−0.92	−0.57	−0.82	−0.82	−0.82	1.35	−0.52	−0.54	−0.73	−0.21	−0.41	−0.40	−0.41
	6-21G**	−0.30	−0.41	−0.64	−0.81	−0.53	−0.64	−0.63	−0.65	−0.18	−0.32	−0.48	−0.65	−0.23	−0.37	−0.36	−0.37
	6-31G**	−0.30	−0.53	−0.80	*	−0.58	−0.76	−0.75	−0.77	−0.25	−0.36	−0.54	−0.70	−0.23	−0.42	−0.40	−0.42
	6-311G**	−0.18	−0.56	−0.84	*	−0.58	−0.77	−0.77	−0.78	−0.04	−0.30	−0.53	−0.69	−0.23	−0.44	−0.42	−0.44
PBE0	cc-pVDZ	−0.25	−0.50	−0.82	*	−0.59	−0.81	−0.81	−0.82	−0.06	−0.21	−0.53	−0.73	−0.23	−0.42	−0.40	−0.42
	cc-pVTZ	0.09	−0.46	−0.88	−0.92	−0.58	−0.81	−0.81	−0.82	0.25	−0.32	−0.54	−0.73	−0.22	−0.42	−0.41	−0.42
	cc-pVQZ	0.48	−0.50	−0.90	−0.92	−0.58	−0.82	−0.82	−0.82	0.62	−0.34	−0.55	−0.74	−0.22	−0.42	−0.40	−0.42
	cc-pV5Z	0.72	−0.65	−0.91	−0.92	−0.58	−0.82	−0.82	−0.83	0.85	−0.37	−0.54	−0.74	−0.21	−0.42	−0.40	−0.42
	aug-cc-pVDZ	−0.50	−0.65	−0.93	−0.94	−0.59	−0.83	−0.83	−0.84	0.08	−0.28	−0.56	−0.72	−0.21	−0.41	−0.40	−0.41
	aug-cc-pVTZ	−0.07	−0.66	−0.92	−0.93	−0.58	−0.82	−0.83	−0.83	0.66	−0.35	−0.55	−0.74	−0.21	−0.42	−0.40	−0.41
	aug-cc-pVQZ	0.33	−0.63	−0.92	−0.93	−0.58	−0.82	−0.83	−0.83	1.13	−0.43	−0.55	−0.74	−0.21	−0.42	−0.40	−0.41
	aug-cc-pV5Z	0.61	−0.91	−0.91	−0.92	−0.58	−0.82	−0.83	−0.83	1.35	−0.54	−0.54	−0.74	−0.21	−0.41	−0.40	−0.41
	6-21G**	−0.33	−0.44	−0.67	−0.83	−0.55	−0.66	−0.66	−0.67	−0.19	−0.34	−0.49	−0.67	−0.23	−0.38	−0.37	−0.38
	6-31G**	−0.32	−0.55	−0.82	*	−0.59	−0.77	−0.77	−0.78	−0.26	−0.36	−0.55	−0.71	−0.24	−0.42	−0.41	−0.42
	6-311G**	−0.20	−0.58	−0.85	*	−0.59	−0.78	−0.78	−0.79	−0.04	−0.30	−0.53	−0.70	−0.23	−0.44	−0.42	−0.44
HF	cc-pVDZ	−0.34	−0.62	−0.89	*	−0.64	−0.86	−0.86	−0.87	−0.07	−0.24	−0.55	−0.77	−0.25	−0.45	−0.43	−0.45
	cc-pVTZ	0.02	−0.57	−0.92	*	−0.64	−0.86	−0.86	−0.86	0.23	−0.35	−0.55	−0.78	−0.24	−0.45	−0.43	−0.45
	cc-pVQZ	0.42	−0.61	−0.93	−0.94	−0.63	−0.86	−0.86	−0.86	0.61	−0.37	−0.55	−0.79	−0.24	−0.44	−0.43	−0.44
	cc-pV5Z	0.69	−0.74	−0.94	−0.94	−0.63	−0.86	−0.86	−0.86	0.85	−0.39	−0.55	−0.79	−0.24	−0.44	−0.43	−0.44
	aug-cc-pVDZ	−0.55	−0.71	−0.95	−0.95	−0.64	−0.87	−0.87	−0.87	0.05	−0.32	−0.57	−0.76	−0.23	−0.44	−0.43	−0.44
	aug-cc-pVTZ	−0.11	−0.77	−0.94	−0.94	−0.63	−0.86	−0.86	−0.86	0.64	−0.37	−0.56	−0.78	−0.23	−0.44	−0.43	−0.44
	aug-cc-pVQZ	0.30	−0.82	−0.94	−0.94	−0.63	−0.86	−0.86	−0.86	1.12	−0.43	−0.55	−0.79	−0.23	−0.44	−0.43	−0.44
	aug-cc-pV5Z	0.60	−1.01	−0.94	−0.94	−0.63	−0.86	−0.86	−0.86	1.35	−0.52	−0.55	−0.79	−0.23	−0.44	−0.43	−0.44
	6-21G**	−0.46	−0.59	−0.78	*	−0.63	−0.75	−0.75	−0.76	−0.21	−0.38	−0.51	−0.89	−0.25	−0.41	−0.40	−0.41
	6-31G**	−0.41	−0.66	−0.88	*	−0.65	−0.83	−0.83	−0.84	−0.27	−0.40	−0.57	−0.75	−0.25	−0.45	−0.44	−0.45
	6-311G**	−0.28	−0.69	−0.91	*	−0.64	−0.84	−0.84	−0.85	−0.06	−0.32	−0.54	−0.74	−0.25	−0.46	−0.45	−0.46

*AIM, requires more flexibility in the basis set than permitted by the smallest of the basis sets considered for LiF.

**TABLE 2:** Comparison of charges obtained for the most polar (LiF) and least polar (HF) diatomic molecules, using MP2 with different population analysis schemes and basis sets.

		Fluorine charge in LiF (ΔEN = 3.00)								Fluorine charge in HF (ΔEN = 1.88)							
		Q_LPA_	Q_MPA_	Q_NPA_	Q_AIM_	Q_HPA_	Q_MK_	Q_CHELP_	Q_CHELPG_	Q_LPA_	Q_MPA_	Q_NPA_	Q_AIM_	Q_HPA_	Q_MK_	Q_CHELP_	Q_CHELPG_
MP2	cc-pVDZ	−0.25	−0.50	−0.86	*	−0.58	−0.82	−0.82	−0.87	−0.06	−0.22	−0.52	−0.73	−0.23	−0.42	−0.41	−0.42
	cc-pVTZ	0.09	−0.47	−0.92	−0.92	−0.58	−0.82	−0.82	−0.86	0.25	−0.32	−0.53	−0.74	−0.22	−0.42	−0.40	−0.42
	cc-pVQZ	0.47	−0.52	−0.94	−0.93	−0.58	−0.83	−0.83	−0.86	0.62	−0.34	−0.54	−0.76	−0.22	−0.42	−0.40	−0.42
	cc-pV5Z	0.72	−0.67	−0.95	−0.93	−0.58	−0.83	−0.83	−0.86	0.85	−0.34	−0.53	−0.76	−0.21	−0.42	−0.40	−0.42
	aug-cc-pVDZ	−0.50	−0.65	−0.97	−0.94	−0.58	−0.84	−0.84	−0.85	0.08	−0.27	−0.56	−0.72	−0.21	−0.42	−0.40	−0.41
	aug-cc-pVTZ	−0.07	−0.71	−0.96	−0.93	−0.58	−0.84	−0.84	−0.85	0.66	−0.34	−0.55	−0.75	−0.21	−0.42	−0.40	−0.41
	aug-cc-pVQZ	0.33	−0.75	−0.95	−0.93	−0.58	−0.84	−0.84	−0.86	1.13	−0.39	−0.55	−0.76	−0.21	−0.42	−0.40	−0.41
	aug-cc-pV5Z	0.61	−0.93	−0.95	−0.93	−0.58	−0.84	−0.84	−0.86	1.35	−0.50	−0.53	−0.76	−0.21	−0.42	−0.40	−0.41
	6-21G**	−0.34	−0.46	−0.74	−0.84	−0.55	−0.67	−0.67	−0.74	−0.19	−0.36	−0.49	−0.69	−0.23	−0.39	−0.38	−0.39
	6-31G**	−0.33	−0.56	−0.86	*	−0.59	−0.78	−0.78	−0.82	−0.26	−0.37	−0.54	−0.72	−0.23	−0.43	−0.42	−0.43
	6-311G**	−0.20	−0.59	−0.89	*	−0.59	−0.79	−0.79	−0.83	−0.04	−0.30	−0.52	−0.70	−0.23	−0.44	−0.42	−0.44

*AIM, requires more flexibility in the basis set than permitted by the smallest of the basis sets considered for LiF.

#### 3.1.2 Wavefunction population analysis

**FIGURE 4:**
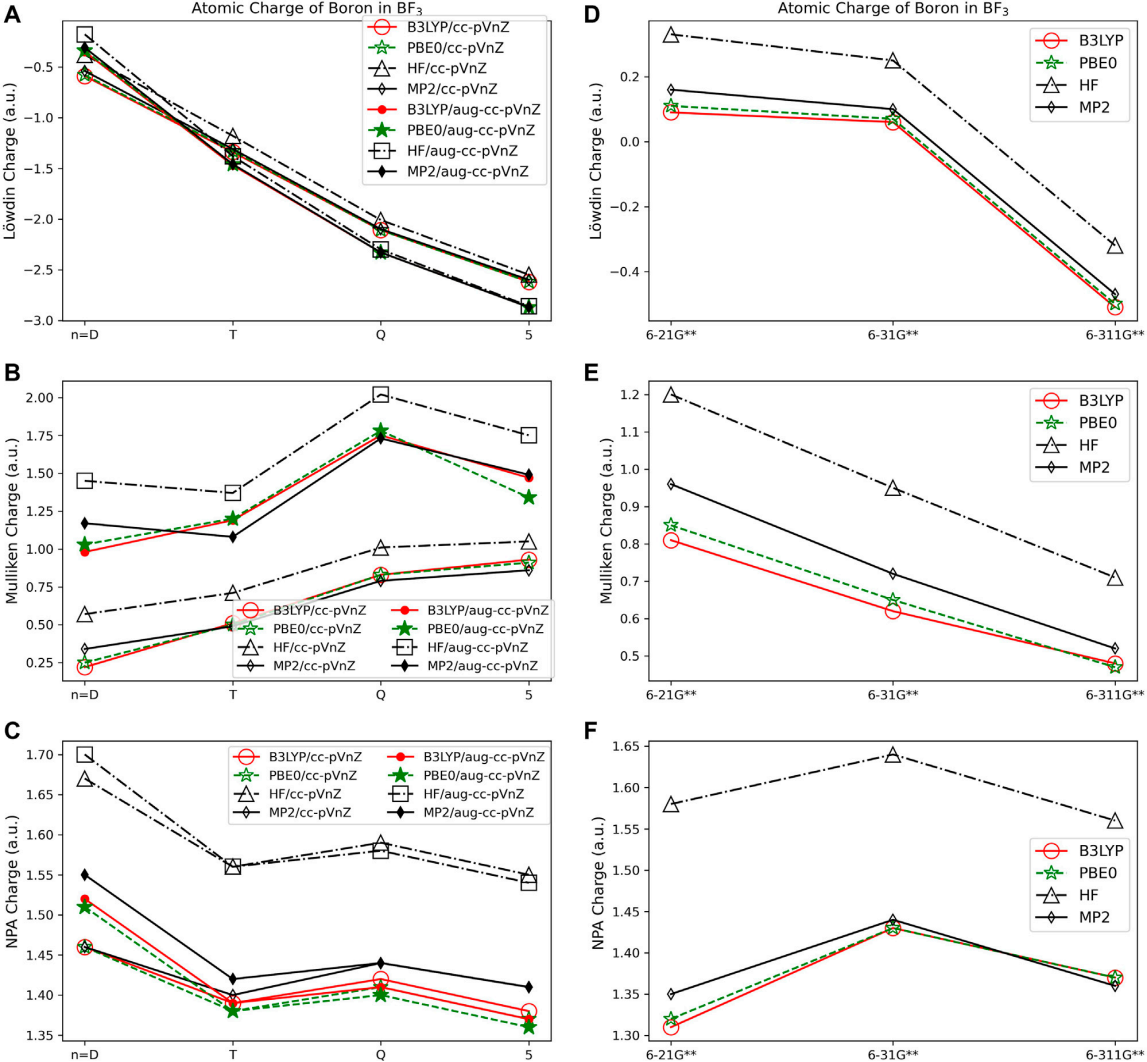
Atomic charge of boron in BF_3_ determined using a number of different theoretical methods, Dunning style basis sets, and wavefunction-based population analysis methods: **(A)** Löwdin, **(B)** Mulliken, and **(C)** NPA. (*Note that scales vary from one analysis method to another.) Pople style basis sets are presented in **(D)** Löwdin, **(E)** Mulliken, and **(F)** NPA.

For population analysis methods, ideally, charge assignment should be independent of basis set and level of theory. In reality, the charge can vary significantly with respect to basis set choice for certain population analysis methods as already noted for HF and LiF. The wavefunction based population analysis methods, Löwdin and Mulliken, have the largest basis set sensitivity as indicated by the large span of assigned charges as shown in Figures 1–3. This basis set sensitivity of Mulliken and Löwdin analysis methods is consistent across the molecules investigated, with the largest basis set effect occurring for the boron charge assigned in BF_3_ using the Löwdin population analysis method, which varies from −2.86 *e* when using HF/aug-cc-pV5Z to 0.33 *e* using HF/6-211G**. In fact, Löwdin charges have the largest basis set dependence for the main group molecules investigated, with a difference in charge as large as 3.19 *e* as for BF_3_. Similar trends in basis set dependence are shown for *ab initio* and DFT methods using Mulliken and NPA schemes (Figure 4). Charges obtained using HF are shown to be consistently larger than the charges assigned using B3LYP, PBE0, and MP2 methods.

Interestingly, despite the electronegativity of fluorine, the Löwdin approach resulted in the assignment of a negative charge to boron when the correlation consistent basis sets were utilized, and for the largest of the Pople style basis sets, 6-311G**. And, in fact, Löwdin charges tend to be unphysical for most of the systems investigated, resulting in negative charges on the cation or on the less electronegative atoms in the compound, particularly when using the correlation consistent basis sets. The charges become larger if diffuse basis functions are used, particularly when there is overlapping electron density originating from neighboring atoms. Figures 4A, D show that the Löwdin method is more dependent on basis set than on the level of theory used in the assignment of atomic charges. However, for basis sets of similar size, the Löwdin atomic charges are similar. For example, using double-ζ correlation consistent basis sets are consistent with the charges obtained using the Pople basis sets. Generally, charges predicted with the larger augmented correlation consistent basis sets show larger deviations from the charges predicted with the Pople style basis sets. These general impacts of basis set choices upon the Löwdin charges, as demonstrated in Figures 4A, D, occur for all molecules investigated. The difference between Löwdin atomic charge obtained using the augmented and non-augmented correlation consistent basis sets is often large as well. For example, the difference between the Löwdin atomic charge obtained using the augmented and non-augmented cc-pV5Z basis sets is as large as 0.89 *e*, as shown for CH_4_ (Supplementary Figure S1). Six compounds (CH_4_, NH_3_, H_2_O, BeCl_2_, MgCl_2_, and HF), have over a 0.44 *e* atomic charge difference between the cc-pV5Z and aug-cc-pV5Z calculations when using the Löwdin population analysis method.

The Mulliken approach resulted in the second widest span of atomic charge predictions for the compounds. As shown in Figure 4B, the Mulliken atomic charge for boron in BF_3_ has a maximum difference of ∼1.80 *e* for B3LYP/cc-pVDZ and HF/aug-cc-pVQZ, and displays a large basis set dependence. However, unlike the Löwdin approach, the Mulliken approach resulted in a positive charge on the boron, which is to be expected for bonding with the electronegative fluorine atoms. The largest span of charges determined by Mulliken population analysis is for CH_4_, with the largest difference of 2.50 *e* occurring for carbon between the MP2/aug-cc-pVTZ (q_C_ = −1.25 *e*) and B3LYP/aug-cc-pVDZ (q_C_ = 1.26 *e*) predictions. Similar as for the Löwdin charges, the Mulliken charges are less basis set dependent when using the smaller Pople style basis sets as compared to the correlation consistent basis sets. This is likely due to the smaller changes between sizes of the Pople basis sets as compared to those of the correlation consistent basis sets with respect to increasing basis set size. It is also important to note that while the correlation consistent basis sets are systematically constructed to converge with increasing basis set quality for energies, this convergence behavior is not necessarily expected for charges. The Mulliken charges do not converge, yet still vary with changes in basis set quality. This non-convergence of Mulliken charges when using correlation consistent basis sets was noted previously, as demonstrated by Martin and Zipse for the Mulliken charge of oxygen on H_2_O (Martin and Zipse, 2005).

Of the orbital-based population analysis methods, natural population analysis is the most consistent in the assignment of atomic charge regardless of the basis set or method used with the exception of the HF method. To illustrate, Figure 4C shows the atomic charges for the boron in BF_3_ as determined using NPA. NPA results in appropriate signs for the atomic charges for all of the molecules. The NPA population analysis method results in charge differences of up to 0.39 *e* for boron in BF_3_ when HF/aug-cc-pVDZ (q_c_ = 1.70) and B3LYP/6-21G**(q_c_ = 1.31) were used. The largest atomic charge span between NPA charges was 0.71 *e* for magnesium in MgO, comparing MP2/aug-cc-pVQZ (q_c_ = 1.48) and B3LYP/6-21G** (q_c_ = 0.77). For MgO, MP2 gives a charge on Mg that is closer to the expected Mg charge of 2+. In Figure 4C the variation in atomic charge is more method dependent than basis set dependent with distinct differences in charges obtained utilizing MP2, PBE0, and B3LYP as compared to those using HF. The NPA atomic charge is higher for cations and lower for anions when utilizing HF as compared to using MP2 or a DFT approach for all of the compounds.

#### 3.1.3 Volume based population analysis

**FIGURE 5:**
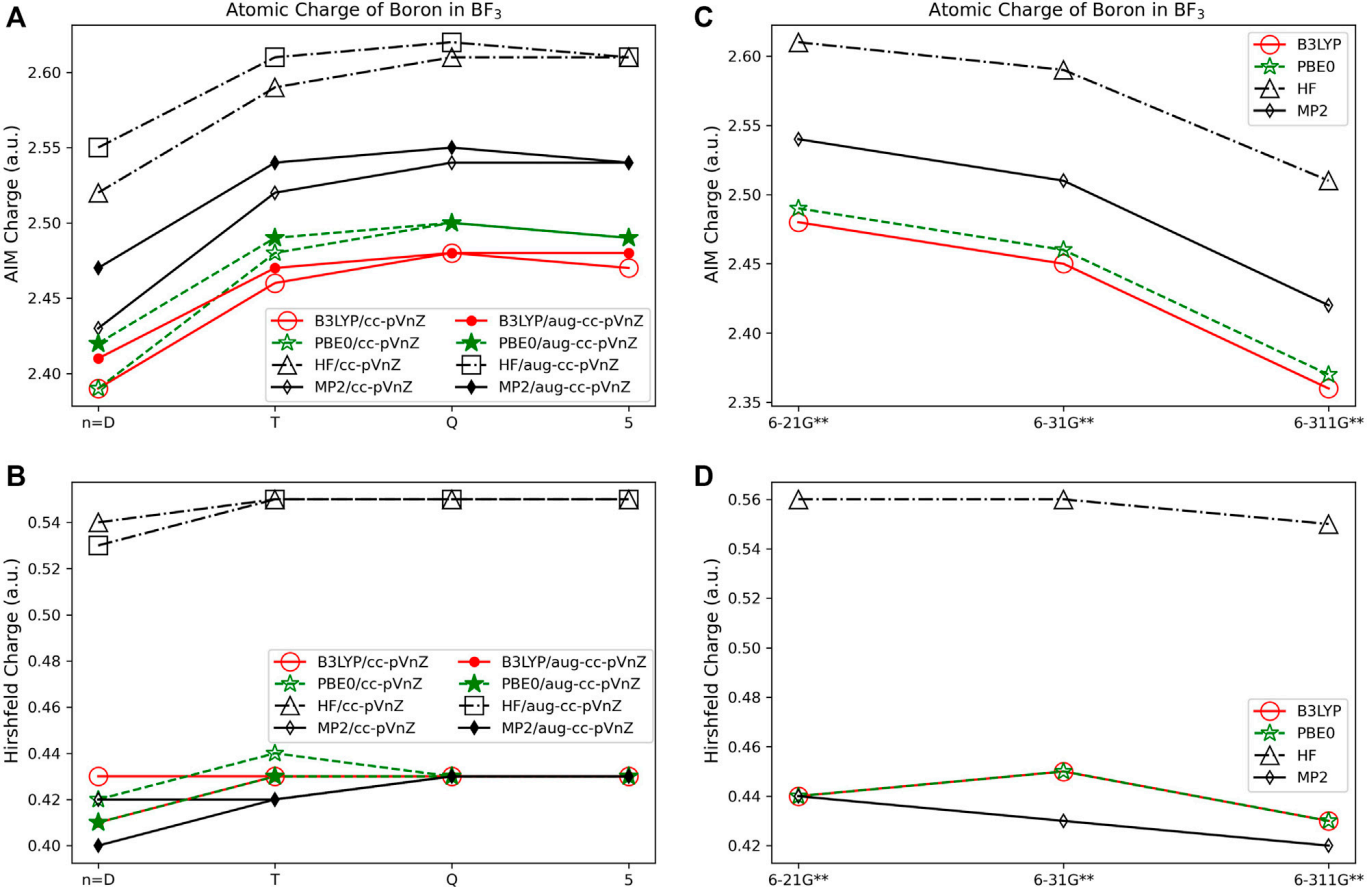
Atomic charge of boron in BF_3_ determined using a number of theoretical methods, Dunning style basis sets, and population analysis methods: **(A)** AIM, and **(B)** Hirshfeld. Pople style basis sets are presented using **(C)** AIM, and **(D)** Hirshfeld.

The charges assigned with volume-based methods are less dependent on basis set and quantum method used as compared to the charges obtained using the Löwdin and Mulliken orbital based methods. The AIM approach results in a higher atomic charge for all cations, and lower atomic charge for all anions in comparison to the other population analysis methods (see, for example, LiF in Figure 1). For the atomic charge of boron determined using AIM for BF_3_ (Figure 5A) there is a ∼0.14 *e* difference between what is predicted with the HF and DFT methods when using the same basis set type; the charges obtained with MP2 fall in between the charges obtained using HF and the DFT methods. The largest AIM charge differences occur for carbon in CO_2_, with a 0.60 *e* difference between the predictions from B3LYP/6-311G** (q_C_ = 2.13 *e*) and HF/aug-cc-pVQZ (q_C_ = 2.73 *e*). When using AIM, charge assignment is less basis set dependent when using the correlation consistent basis sets *versus* the Pople style basis sets.

In contrast to the Löwdin and Mulliken population analysis methods, Hirshfeld is the most consistent overall with charge assignment having little dependence on the basis set and theoretical method. The Hirshfeld method results in differences in the calculated charges of less than or equal to 0.05 *e* regardless of the chosen basis set or theoretical method utilized for five of the molecules investigated, while three main group compounds investigated have differences in atomic charge greater than 0.12 *e*. Investigating the small differences in assigned atomic charge, Hirshfeld charges are shown to depend slightly more on the choice of theoretical method than on the quality of basis set. In Figure 5B, for example, the boron charge in BF_3_ using the Hirshfeld method differs by no more than ∼0.15 *e* between methods. HF theory assigns larger charges (in absolute value) to boron in BF_3_ than MP2, B3LYP, and PBE0, independent of basis set. This same trend occurs for all of the molecules. In Figure 5 it is shown that basis set variations are nearly eliminated for all but the smallest basis sets, though there is a slightly larger difference between the charges obtained using the Pople basis sets with MP2, as compared to charges obtained using these sets with HF or DFT.

#### 3.1.4 Electrostatic potential based population analysis

**FIGURE 6:**
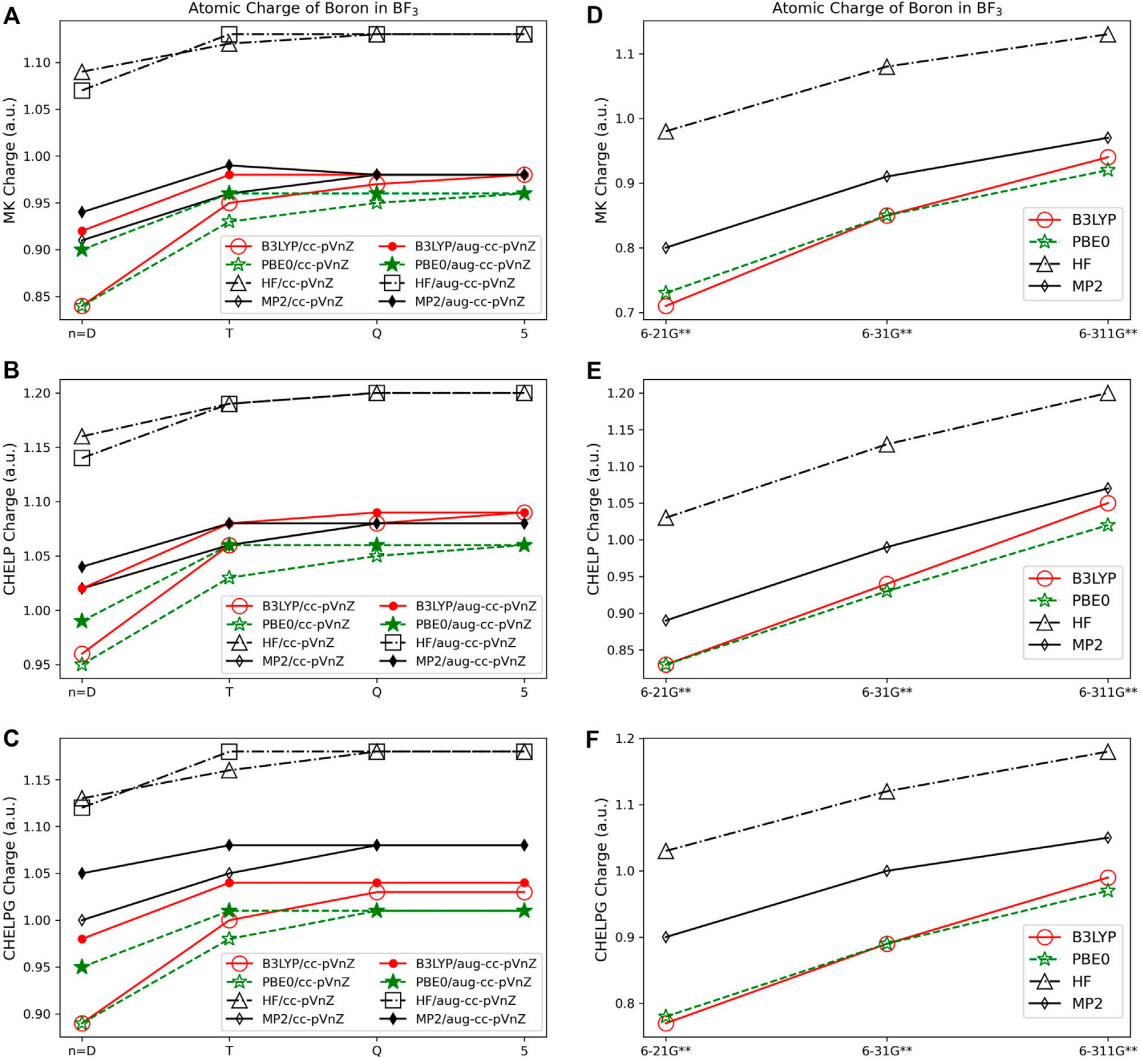
Atomic charge of boron in BF_3_ determined using a number of theoretical methods, basis sets, and population analysis methods: **(A)** Merz-Kollman, **(B)** CHELP, and **(C)** CHELPG. Pople style charges are presented in **(D)** Merz-Kollman, **(E)** CHELP, and **(F)** CHELPG.

Figures 6A–F show the boron charge in BF_3_ for Merz-Kollman, CHELP, and CHELPG population analysis schemes. It is important to note that previous work has shown that the Merz-Kollman and CHELP have a dependence on molecular orientation, on average 0.04–0.05 *e*, and CHELPG has a minimal 0.001 *e* dependence upon the rotation of the molecule (Sigfridsson and Ryde, 1998). The charges of the molecule vary by the same magnitude, regardless of the quantum mechanical method, basis set, and molecule examined with these analysis schemes, with the largest charge span of 0.47 *e* for BF_3_, and an average atomic charge span of 0.39 *e* considering all quantum methods and basis sets. The three electrostatic potential methods result in similar charge assignments. While electrostatic population analysis methods are less dependent on quantum method and basis set used than Löwdin and Mulliken methods, they are slightly more dependent on quantum method and basis set used than the NPA, Hirshfeld, and AIM population analysis methods. Using HF the magnitude of the charge is increased as compared to the charge from B3LYP and PBE0, with the charges obtained using MP2 often falling between HF and DFT, as for the AIM charges. Charges assigned when using the correlation consistent basis sets show even less variation than the Pople sets when the basis set size is increased (see Figure 6). It is also shown that the CHELP atomic charge for carbon in CH_4_ is predicted to be positive for most basis set and quantum method combinations (see Supplementary Figure S1). All other ESP methods predict negative values for carbon along with the most numerically stable population analysis method, Hirshfeld. Based on these considerations and the afore mentioned performance of CHELP as compared to CHELPG (Sigfridsson and Ryde, 1998), CHELP is not recommended for atomic charge.

### 3.2 Vanadium oxide (VO)

**FIGURE 7:**
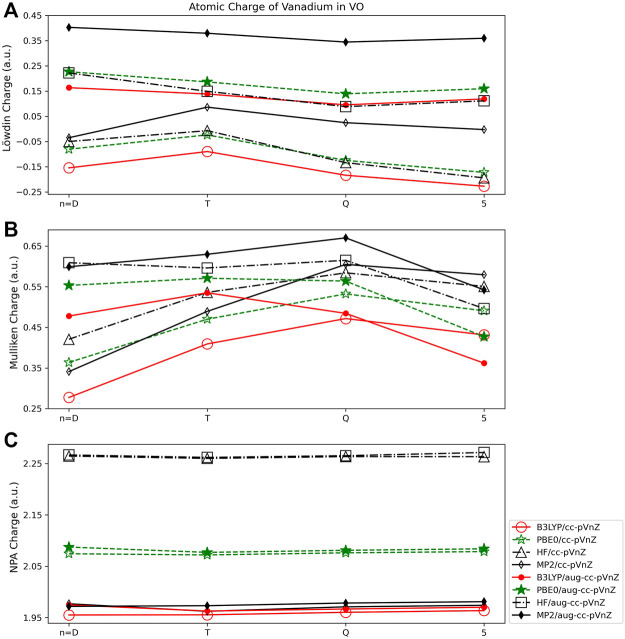
Atomic charge of vanadium in VO determined using a number of different theoretical methods, Dunning style basis sets, and wavefunction-based population analysis methods: **(A)** Löwdin, **(B)** Mulliken, and **(C)** NPA (*Note that scales vary from one analysis method to another.).

To consider a transition metal, vanadium oxide (VO) was investigated. All charges obtained for VO are given in the SI (Supplementary Tables S13, S14). The Mulliken populations obtained in this work (Figure 7B) are consistent with prior work by Miliordos and Mavridis. In their prior extensive study of the electronic structure of the VO molecule, where both its neutral and cationic/anionic forms were examined (Miliordos and Mavridis, 2007) using multireference and coupled cluster methods, Mulliken population analysis indicated an electron transfer of ∼0.5 *e* from the V atom to the O atom in neutral VO.

#### 3.2.1 Wavefunction population analysis

For vanadium oxide, the Lӧwdin population analysis showed less dependence on the size of the basis set than was observed for main group molecules. For example, the largest difference between basis sets for a single quantum method using the Lӧwdin population scheme is for PBE0, where an absolute charge difference of 0.149 *e* is observed between the cc-pVTZ and cc-pV5Z basis sets (Figure 7A). This is in contrast to an example from the main group species, where for MgO, the difference in Lӧwdin charge obtained using the cc-pVDZ and cc-pV5Z basis sets is markedly larger, at ∼0.5 *e*. However, it should be noted that for several of the calculations, the Lӧwdin population analysis predicted a negative charge on the vanadium atom when non-augmented basis sets were used. This indicates that the diffuse flexibility in the basis set is important for obtaining charges that are physically meaningful (positive charges on metal atoms), at least for VO. In addition, the Löwdin method displayed a considerable dependence on the quantum method used, with the largest charge difference of 0.25 *e* occurring between the HF (0.11 *e*) and MP2 (0.36 *e*) determinations with the aug-cc-pVQZ basis set.

The basis set size dependence of the Mulliken population analysis (Figure 7B) for VO is similar to that of HF and LiF. For example, the difference in Mulliken charge obtained for the HF molecule using the B3LYP functional was 0.16 *e* (using the cc-pVDZ and cc-pV5Z basis sets), and for LiF using B3LYP the difference in charge obtained using the same basis sets was 0.17 *e*. For the same method and basis sets the charge difference in VO is 0.15 *e*. For the augmented correlation consistent basis sets however, the Mulliken charges vary less with basis set size as compared to the charges arising from use of the non-augmented sets for VO, where the opposite was observed for the HF and LiF molecules. The Mulliken charges obtained using B3LYP and the aug-cc-pVDZ and aug-cc-pV5Z sets for VO differ by just 0.12 *e*, for example, while for the HF molecule using the same basis sets and quantum method the difference is 0.27 *e*. Mulliken population analysis shows slightly less dependence on the quantum method as compared to the Löwdin method when applied to VO, with a maximum difference of 0.19 *e* between the charges obtained using aug-cc-pVQZ in combination with the B3LYP (0.48 *e*) and MP2 (0.67 *e*) methods.

As demonstrated for the main group molecules, of the wavefunction based population schemes, NPA shows much less dependence on basis set than either the Lӧwdin or Mulliken schemes (Figure 7C). Additionally, unlike for the Lӧwdin or Mulliken schemes, there is very little (0.02 *e* or smaller) difference between the augmented and non-augmented basis sets of the same *n* (for example, the cc-pVDZ and aug-cc-pVDZ basis sets) obtained using the same quantum method. The B3LYP and MP2 results are shown to be quite consistent, varying by 0.02 *e* or less, while the NPA charges obtained using PBE0 are ∼0.12 *e* larger. Finally, the HF results are furthest from the two DFT and the MP2 results, as was observed for main group molecules, resulting in charges of ∼2.26 *e*, regardless of basis set used.

#### 3.2.2 Volume based population analysis

**FIGURE 8:**
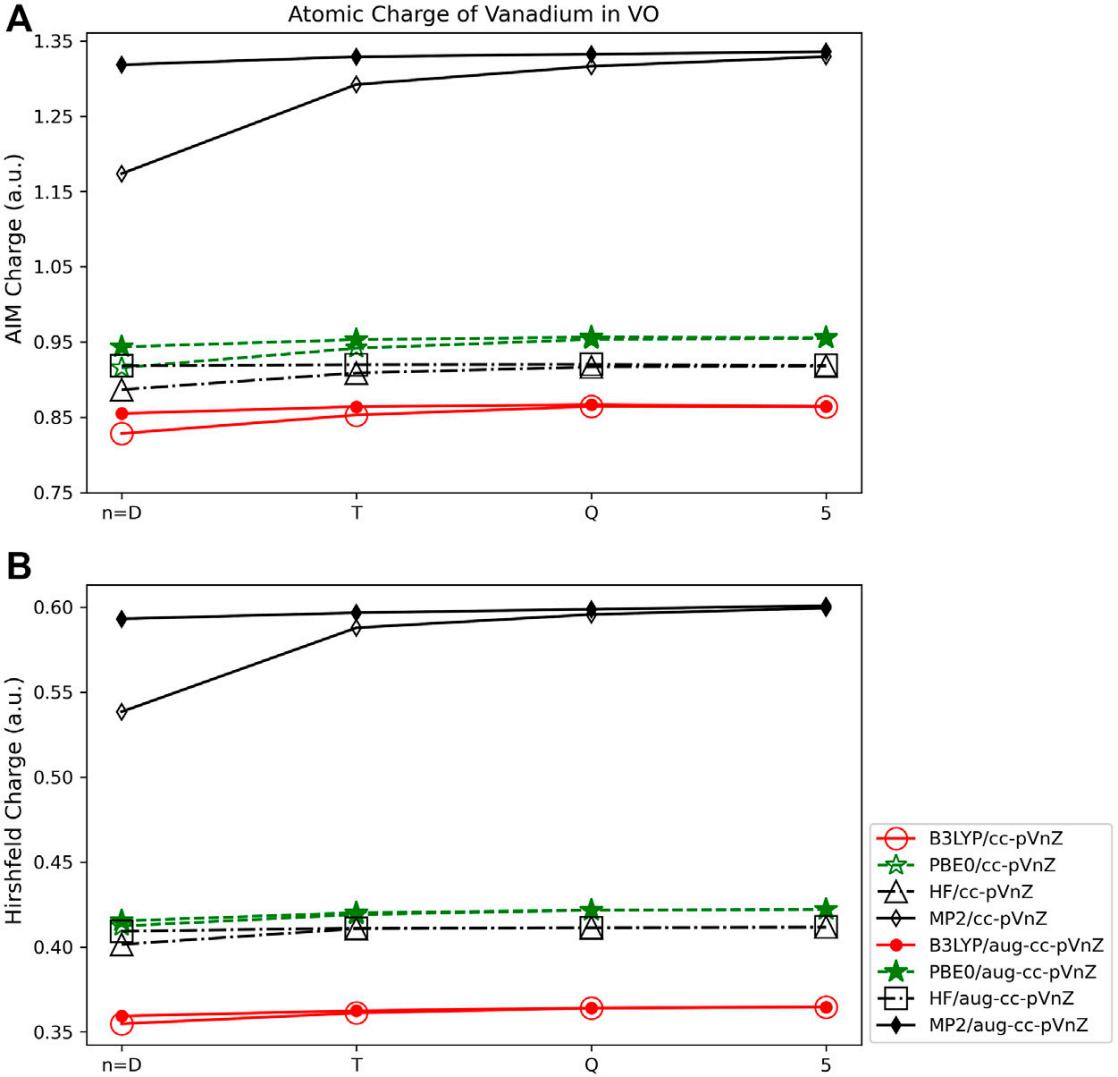
Atomic charge of vanadium in VO determined using a number of theoretical methods, Dunning style basis sets, and population analysis methods: **(A)** AIM, and **(B)** Hirshfeld (*Note that scales vary from one analysis method to another.).

As was observed for the main group molecules, the AIM and Hirshfeld charges show much less basis set and quantum method dependance than the Mulliken and Lӧwdin wavefunction based methods. The overall basis set level or addition of augmented functions affects the charge very little, usually by ∼0.01 *e* or less for any particular quantum method. Though the volume-based methods are in general less dependent on quantum method, from Figure 8 it is clear that the MP2 method results in charges that are less consistent with the HF, B3LYP, and PBE0 results. This is in contrast to the observations in population method trends for the main group species, where HF charges tended to be the least consistent as compared to the charges arising from the other three quantum methods (see, for example, Figure 5). Similar as for main group species, however, is that the Hirshfeld charges are the most independent of basis set or quantum method used, varying by ∼0.25 *e* at most (Figure 8B).

#### 3.2.3 Electrostatic potential based population analysis

**FIGURE 9:**
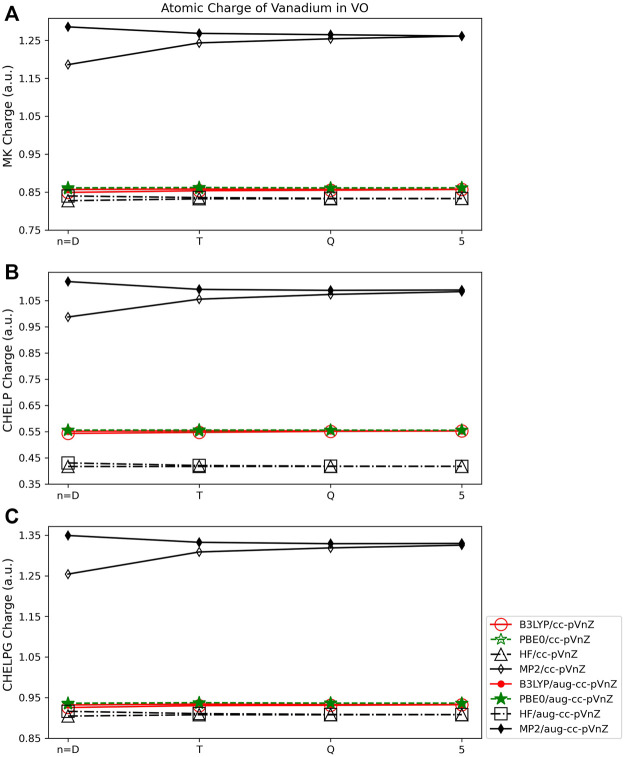
Atomic charge of vanadium in VO determined using a number of theoretical methods, basis sets, and population analysis methods: **(A)** Merz-Kollman, **(B)** CHELP, and **(C)** CHELPG (*Note that scales vary from one analysis method to another.).

The Merz-Kollman (MK) and CHELPG population analysis methods when applied to the VO molecule (Figure 9) show similar dependence on quantum method as the Mulliken and AIM approaches. The MK and CHELPG methods are less dependent on quantum method than the Lӧwdin method, and more so than either the Hirshfeld or NPA approaches. The largest span between charges obtained using MK and CHELPG are both ∼0.45 *e* when all basis sets and methods are considered. The CHELP method on the other hand shows much more dependence on quantum method, with a maximum charge difference of ∼0.70 (Figure 9B). All three ESP approaches show very little basis set dependence, however, both when the relative size of the basis set and augmented and non-augmented sets are compared. The largest basis set difference is between the cc-pVDZ and aug-cc-pVDZ basis sets when using the MP2 quantum approach (for all three ESP methods). For example, the cc-pVDZ/MK charge is 1.19 *e*, and the aug-cc-pVDZ/MK charge is 1.29 *e*. This difference is shown in Figures 9A–C. For all basis sets other than the double-ζ set, however, the effect of different basis sets for a given quantum method is very small, on the order of 0.01–0.02 *e* for all three ESP methods.

### 3.3 Lawrencium fluoride (LrF)

**FIGURE 10:**
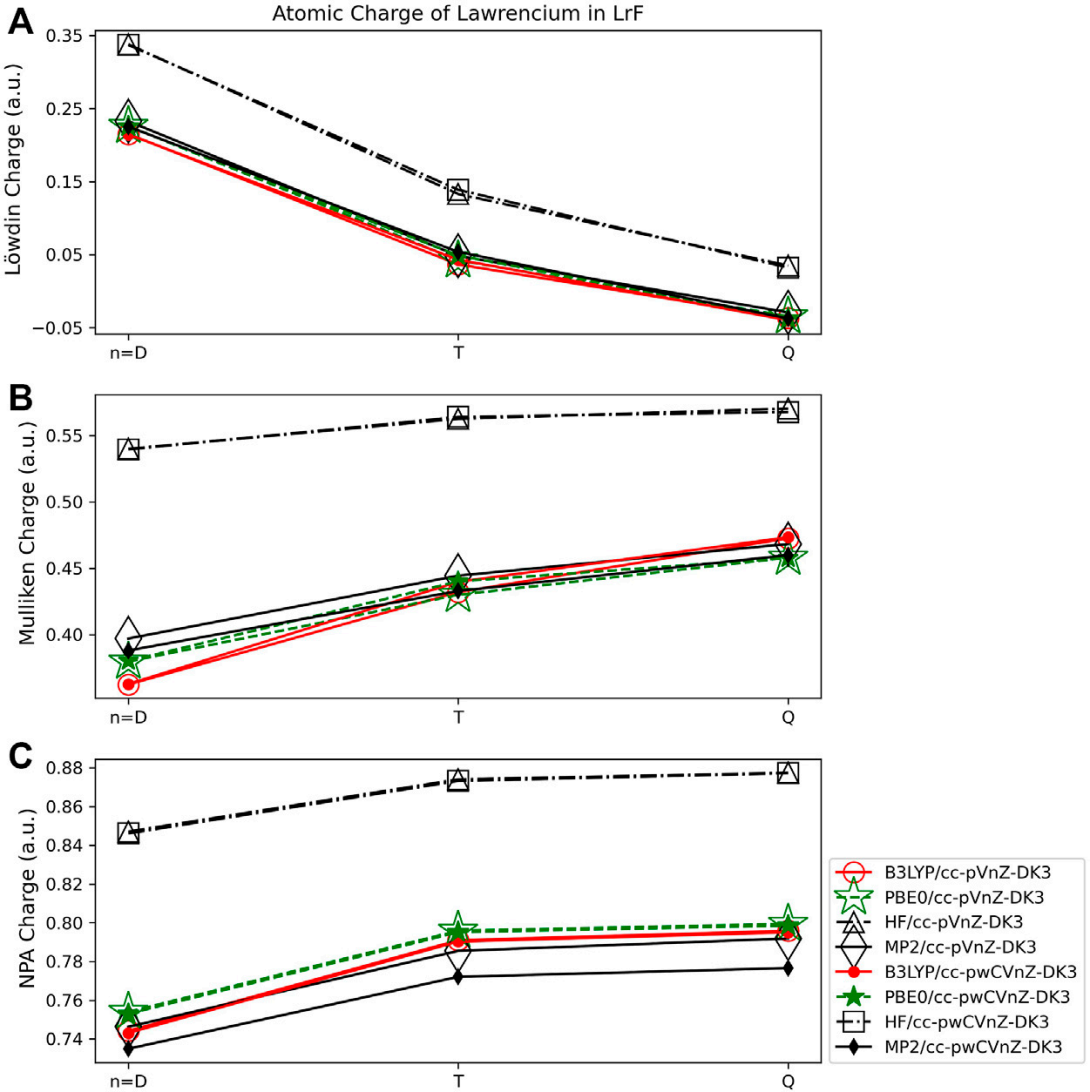
Atomic charge of lawrencium in LrF determined using a number of different theoretical methods, Dunning style basis sets, and wavefunction-based population analysis methods: **(A)** Löwdin, **(B)** Mulliken, and **(C)** NPA (*Note that scales vary from one analysis method to another.).

The lawrencium fluoride molecule was chosen to provide insight about the behavior of population analysis on an actinide-containing molecule. All charges obtained for the LrF molecule are given in Supplementary Tables S15, S16 in the SI. There are limited studies of LrF, however, Laerdahl et al. (1998) did calculate the Mulliken population on the Lr atom in LrF using the Dirac-Hartree-Fock (DHF) method and an author-generated gaussian basis set, giving a total charge on the metal of 0.86 *e*. This charge is approximately 0.30 *e* larger than that found in this work using HF/cc-pVQZ-DK3 (Figure 10A). The ground state electronic structure for this molecule is a closed-shell singlet (^1^Σ^+^) that is well separated (∼22,225 cm^-1^) from the first excited state, according to recent work done using the multireference configuration interaction method with a Davidson correction (MRCI + Q) (North et al., 2023). For heavy element species, it is important to include relativistic effects in both the basis set and Hamiltonian. The cc-pV*n*Z-DK3 and cc-pwCV*n*Z-DK3 basis sets with *n* = D,T,Q (Peterson, 2015; Feng and Peterson, 2017) were contracted for the third-order Douglas-Kroll-Hess (DKH3) relativistic Hamiltonian (Reiher and Wolf, 2004). This is one of the first, if not the first time that these basis sets have been examined with respect to population analysis methods.

#### 3.3.1 Wavefunction population analysis

The charges on the Lr atom obtained using the Löwdin method are shown to be more dependent on the size of the basis set used than for VO. The charges obtained using HF show the largest basis set dependence for this population scheme. Here, the charge obtained using HF/cc-pVDZ-DK3 is 0.34 *e*, while that obtained using HF/cc-pVQZ-DK3 is 0.03 *e*. This is still a smaller difference, however, than was observed for main-group diatomics, such as MgO which exhibited a charge difference of ∼0.5 *e* depending on basis set used, and LiF which showed an even larger basis set dependence of ∼1.25 *e*. The difference between the cc-pV*n*Z-DK3 and cc-pwCV*n*Z-DK3 basis sets is shown to be very small for this and all other population methods, amounting to differences of charge of 0.001–0.01 *e* for basis sets with the same cardinal number (such as double-ζ). As was observed for VO, the Löwdin method results in negative charges for the Lr atom when some basis sets are used (cc-pVQZ-DK3 and cc-pwCVQZ-DK3). Regarding quantum method dependence, the Löwdin charges obtained using MP2, B3LYP, and PBE0 are all within 0.01 *e* of each other at any given basis set level. The HF method results in Löwdin charges that are ∼0.10 *e* larger than those obtained using the other three methods.

For LrF, the Mulliken population method shows less basis set dependence than the Löwdin populations. The largest difference in Mulliken charges are those obtained using B3LYP, where the charge difference is still only 0.11 *e* (Figure 10B). This is similar as for VO and for main group diatomics. The Mulliken charges obtained for LrF exhibit similar quantum method dependence as for Lowdin charges, with MP2 and DFT results being consistent with each other, and HF charges larger; in this case, the difference between the charges obtained using the HF method and the other three quantum methods is slightly larger (∼0.15 *e*).

Finally, the charges obtained using NPA for LrF (Figure 10C) are again the least basis set dependent on the wavefunction methods, with the largest difference being 0.06 *e* for the charges obtained using the cc-pVDZ-DK3 and cc-pVQZ-DK3 basis sets and B3LYP. The charges determined using NPA show similar quantum method dependence as for the Löwdin and Mulliken methods, with MP2 and both DFT methods yielding charges within ∼0.01 *e* of one another at any given basis set level, and the HF charges being ∼0.10 *e* larger than those obtained with the other three quantum methods.

#### 3.3.2 Volume based population analysis

**FIGURE 11:**
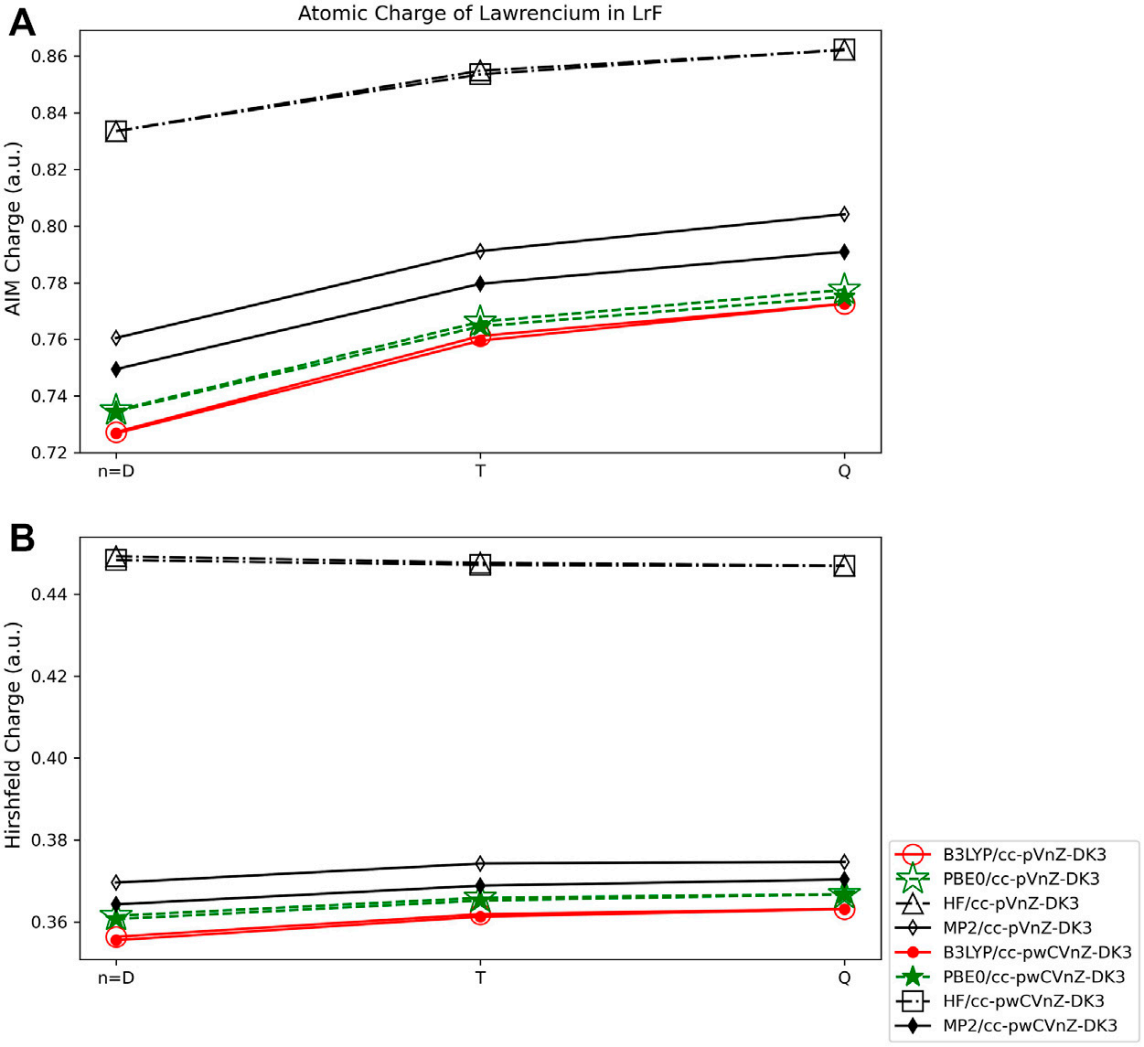
Atomic charge of lawrencium in LrF determined using a number of theoretical methods, Dunning style basis sets, and population analysis methods: **(A)** AIM, and **(B)** Hirshfeld (*Note that scales vary from one analysis method to another.).

The volume-based population methods applied to LrF show limited basis set dependence, with charges obtained using the AIM method (Figure 11A) varying by 0.05 *e* at most, such as those obtained using the PBE0 functional and double-ζ and quadruple-ζ basis sets. The charges obtained using the Hirshfeld method (Figure 11B) are even less dependent on the basis set used, with charges varying by less than 0.01 *e* for any given quantum method. The DFT and MP2 methods result in charges that are within 0.01 *e* of one another at any basis set level, with charges obtained using HF ∼0.10 *e* larger than the other quantum methods.

## 4 Conclusion

In this work, the effect of basis set and quantum mechanical method choices on the prediction of atomic charges with eight different population analysis schemes was investigated. Tables 3, 4 summarize the data and conclusions drawn for each of the population analysis methods considered herein, for main group molecules, a transition metal molecule (VO), and heavy metal molecule (LrF). Wavefunction-based, volume-based, and ESP methods were utilized. Molecules including main group, transition metal, and heavy element species were considered. This is one of the first, if not the first time the cc-pV*n*Z-DK3 and cc-pwCV*n*Z-DK3 basis sets for an actinide molecule have been utilized in population analysis. The largest basis set dependence for the LrF molecule was observed when the Lӧwdin population analysis was used, with the HF method, where the charges varied by as much as 0.31 *e*, depending upon basis set chosen.

**TABLE 3:** Basis set and quantum method dependencies from wavefunction and volume based population analysis methods applied to main group, transition metal and heavy metal molecules.

Scheme type	Population method	Main group	Transition metal	Heavy metal
Wavefunction/orbital based	Mulliken	large basis set sensitivity	similar as for main group	similar as for main group
Wavefunction/orbital based	Löwdin	largest basis set sensitivity, non-physical charge assignment	basis set dependence less than observed for main group	more dependent on basis set when used for LrF than VO, but less basis set dependent than when used for main group
Wavefunction/orbital based	NPA	least basis set dependent of the wavefunction based methods	least basis set dependent of the wavefunction based methods	least basis set dependent of the wavefunction based methods
Volume based	AIM	less dependent on basis set and method than wavefunction based methods	less dependent on basis set and method than wavefunction based methods	less dependent on basis set and method than wavefunction based methods
Volume based	Hirshfeld	least dependent on basis set/quantum method of population schemes considered	least dependent on basis set/quantum method of population schemes considered	least dependent on basis set/quantum method of population schemes considered

**TABLE 4:** Basis set and quantum method dependencies from electrostatic potential population analysis methods applied to main group and transition metal molecules.

Scheme type	Population method	Main group	Transition metal
ESP	Chelp	not recommended: assigns non-physical charges	large dependence of quantum method
ESP	ChelpG	more dependent on quantum method and basis set than NPA, Hirshfeld, and AIM	more dependent on quantum method than NPA and Hirshfeld
ESP	Merz-Kollman	more dependent on quantum method and basis set than NPA, Hirshfeld, and AIM	more dependent on quantum method than NPA and Hirshfeld

The effect of molecular polarity in the determination of atomic charge was evaluated. It was shown that for more polar molecules, basis set dependence becomes more significant, regardless of quantum method used, especially for the wavefunction based population analysis schemes.

Ideally, charge assignment schemes should be independent of basis set and method approach. The Hirshfeld population analysis atomic charges are the least sensitive to changes in basis set and quantum method as compared to those of the other population analysis methods investigated. However, the Hirshfeld method underestimates the charge on molecules such as LiF, where expected charges are ∼+1 and ∼-1 on the Li and F, respectively due to ionic bonding. AIM most often predicts charges that are expected from ionic bonding, followed closely by NPA. The largest variations in atomic charges occur for the Löwdin population analysis method. Löwdin and CHELP atomic charges can result in unphysical charges with the correlation consistent basis sets or large Pople style basis sets. For the VO molecule, the use of augmented correlation consistent basis sets was found to be important for obtaining physically meaningful charges when the Löwdin method was used.

Orbital-based methods are shown to have a much larger dependence on basis set than electrostatic potential and volume-based methods. Utilizing NPA reduces the basis set dependence compared to other orbital based methods such as Mulliken and Löwdin charges. Volume and potential based methods also typically show a reduction in basis set and quantum method dependency. Out of the volume-based schemes considered, the Hirshfeld method has the least dependence on basis set for the molecule set. Quantum method dependence is also important with maximum differences in charges from ∼0.10 *e* to 0.50 *e* when within a particular basis set and population scheme, especially when smaller basis sets are used. Caution should be taken when using population analysis methods to assign charge as they are often shown to be both basis set and quantum method dependent.

## Data Availability

The original contributions presented in the study are included in the article/Supplementary Material, further inquiries can be directed to the corresponding author.

## References

[B1] Adamo C. Barone V. (1999). Toward reliable density functional methods without adjustable parameters: The PBE0 model. J. Chem. Phys. 110 (13), 6158–6170. 10.1063/1.478522

[B2] Allen F. H. Kennard O. Watson D. G. Brammer L. Orpen A. G. Taylor R. (1987). Tables of bond lengths determined by X-ray and neutron diffraction. Part 1. Bond lengths in organic compounds. J. Chem. Soc. Perkin Trans. 2 (12), S1. 10.1039/p298700000s1

[B3] Bachrach S. M. (1994). “Population analysis and electron densities from quantum Mechanics,” in Reviews in computational chemistry, volume V. Editors Lipkowitz K. B. Boyd D. B. (New York: VCH Publishers, Inc.), 171–227.

[B4] Bader R. F. W. (1990). Atoms in molecules: A quantum theory. Oxford, United Kingdom: Clarendon Press, 438. International series of monographs on chemistry, xviii. 10.1002/0470845015.caa012

[B5] Bai Z. Jiang X. Z. Luo K. H. (2022). Effects of water on pyridine pyrolysis: A reactive force field molecular dynamics study. Energy 238, 121798. 10.1016/j.energy.2021.121798

[B6] Balabanov N. B. Peterson K. A. (2006). Basis set limit electronic excitation energies, ionization potentials, and electron affinities for the 3d transition metal atoms: Coupled cluster and multireference methods. J. Chem. Phys. 125 (7), 074110. 10.1063/1.2335444 16942325

[B7] Balabanov N. B. Peterson K. A. (2005). Systematically convergent basis sets for transition metals. I. All-electron correlation consistent basis sets for the 3d elements Sc-Zn. J. Chem. Phys. 123 (6), 064107. 10.1063/1.1998907 16122300

[B8] Bayly C. I. Cieplak P. Cornell W. Kollman P. A. (1993). A well-behaved electrostatic potential based method using charge restraints for deriving atomic charges: The RESP model. J. Phys. Chem. 97 (40), 10269–10280. 10.1021/j100142a004

[B9] Besler B. H. Merz K. M. Kollman P. A. (1990). Atomic charges derived from semiempirical methods. J. Comput. Chem. 11 (4), 431–439. 10.1002/jcc.540110404

[B10] Binkley J. S. Pople J. A. (1977). Self‐consistent molecular orbital methods. XIX. Split‐valence Gaussian‐type basis sets for beryllium. J. Chem. Phys. 66 (2), 879–880. 10.1063/1.433929

[B11] Breneman C. M. Wiberg K. B. (1990). Determining atom-centered monopoles from molecular electrostatic potentials. The need for high sampling density in formamide conformational analysis. J. Comput. Chem. 11 (3), 361–373. 10.1002/jcc.540110311

[B12] Bruhn G. Davidson E. R. Mayer I. Clark A. E. (2006). Löwdin population analysis with and without rotational invariance. Int. J. Quantum Chem. 106 (9), 2065–2072. 10.1002/qua.20981

[B13] Bultinck P. Van Alsenoy C. Ayers P. W. Carbó-Dorca R. (2007). Critical analysis and extension of the Hirshfeld atoms in molecules. J. Chem. Phys. 126 (14), 144111. 10.1063/1.2715563 17444705

[B14] Catone D. Satta M. Castrovilli M. C. Bolognesi P. Avaldi L. Cartoni A. (2021). Photoionization of methanol: A molecular source for the prebiotic chemistry. Chem. Phys. Lett. 771, 138467. 10.1016/j.cplett.2021.138467

[B15] Chirlian L. E. Franclt M. M. Francl M. M. (1987). Atomic charges derived from electrostatic potentials: A detailed study. J. Comput. Chem. 8 (6), 894–905. 10.1002/jcc.540080616

[B16] Cho M. Sylvetsky N. Eshafi S. Santra G. Efremenko I. Martin J. M. L. (2020). The atomic partial charges arboretum: Trying to see the forest for the trees. ChemPhysChem 21 (8), 688–696. 10.1002/cphc.202000040 32052532 PMC7317385

[B17] Cramer C. J. (2002). Essentials of computational chemistry. 2nd edn. New York: Wiley.

[B18] da Silva T. U. Pougy K. d. C. Albuquerque M. G. da Silva Lima C. H. Machado S. d. P. (2022). Development of parameters compatible with the CHARMM36 force field for [Fe4S4]2+ clusters and molecular dynamics simulations of adenosine-5’-phosphosulfate reductase in GROMACS 2019. J. Biomol. Struct. Dyn. 40 (8), 3481–3491. 10.1080/07391102.2020.1847687 33183173

[B19] Davidson E. R. Chakravorty S. (1992). A test of the Hirshfeld definition of atomic charges and moments. Theor. Chim. Acta 83, 319–330. 10.1007/bf01113058

[B20] Davidson E. R. Clark A. E. (2022). “A viewpoint on population analyses,” in International journal of quantum chemistry (New York, United States: John Wiley and Sons Inc). 10.1002/qua.26860

[B21] Dill J. D. Pople J. A. (1975). Self-consistent molecular orbital methods. XV. Extended Gaussian-type basis sets for lithium, beryllium, and boron. J. Chem. Phys. 62, 2921–2923. 10.1063/1.430801

[B22] Dunning T. H., J. Peterson K. A. Wilson A. K. (2001). Gaussian basis sets for use in correlated molecular calculations. X. The atoms aluminum through argon revisited. J. Chem. Phys. 114 (21), 9244–9253. 10.1063/1.1367373

[B23] Dunning T. H. J. (1989). Gaussian basis sets for use in correlated molecular calculations. I. The atoms boron through neon and hydrogen. J. Chem. Phys. 90 (2), 1007–1023. 10.1063/1.456153

[B24] Feng R. Peterson K. A. (2017). Correlation consistent basis sets for actinides. II. the atoms Ac and Np-Lr. J. Chem. Phys. 147 (8), 084108. 10.1063/1.4994725 28863538

[B25] Foster J. P. Weinhold F. (1980). Natural hybrid orbitals. J. Am. Chem. Soc. 102 (22), 7211–7218. 10.1021/ja00544a007

[B26] Francl M. M. Pietro W. J. Hehre W. J. Binkley J. S. Gordon M. S. DeFrees D. J. (1982). Self-consistent molecular orbital methods. XXIII. A polarization-type basis set for second-row elements. J. Chem. Phys. 77 (7), 3654–3665. 10.1063/1.444267

[B27] Frisch M. J. Trucks G. W. Schlegel H. B. Scuseria G. E. Robb M. A. Cheeseman J. R. (2016). Gaussian 16, revision C.01. Wallingford, CT: Gaussian, Inc.

[B28] Gordon S. Binkley J. S. Pople J. A. Pietro W. J. Hehre W. J. (1982). Self-consistent molecular-orbital methods. 22. Small split-valence basis sets for second-row elements. J. Am. Chem. Soc. 104, 2797–2803. 10.1021/ja00374a017

[B29] Hariharan P. C. Pople J. A. (1973). The influence of polarization functions on molecular orbital hydrogenation energies. Theor. Chim. Acta 28, 213–222. 10.1007/bf00533485

[B30] Hehre W. J. Ditchfield R. Pople J. A. (1972). Self - consistent molecular orbital methods. XII. Further extensions of Gaussian - type basis sets for use in molecular orbital studies of organic molecules. J. Chem. Phys. 56, 2257–2261. 10.1063/1.1677527

[B31] Heidar-Zadeh F. Ayers P. W. Verstraelen T. Vinogradov I. Vöhringer-Martinez E. Bultinck P. (2018). Information-theoretic approaches to atoms-in-molecules: Hirshfeld family of partitioning schemes. J. Phys. Chem. A 122 (17), 4219–4245. 10.1021/acs.jpca.7b08966 29148815

[B32] Hirshfeld F. L. (1977). Bonded-atom fragments for describing molecular charge densities. Theor. Chim. Acta 44 (2), 129–138. 10.1007/BF00549096

[B33] Jabłoński M. Palusiak M. (2010). Basis set and method dependence in quantum theory of atoms in molecules calculations for covalent bonds. J. Phys. Chem. A 114 (47), 12498–12505. 10.1021/jp106740e 21049895

[B34] Jensen F. (2007). Introduction to computational chemistry. 2nd Ed. Chichester: John Wiley & Sons, Ltd. 10.1007/s00214-013-1372-6

[B35] Johnson R. D. III (2015). NIST computational chemistry comparison and Benchmark Database., NIST standard reference, Database number 101.

[B36] Kendall R. A. Dunning T. H. Harrison R. J. (1992). Electron affinities of the first-row atoms revisited. Systematic basis sets and wave functions. J. Chem. Phys. 96 (9), 6796–6806. 10.1063/1.462569

[B37] Kognole A. A. Lee J. Park S. Jo S. Chatterjee P. Lemkul J. A. (2022). CHARMM-GUI Drude prepper for molecular dynamics simulation using the classical Drude polarizable force field. J. Comput. Chem. 43 (5), 359–375. 10.1002/jcc.26795 34874077 PMC8741736

[B38] Laerdahl J. K. Fægri K. Visscher L. Saue T. (1998). A fully relativistic Dirac – Hartree – Fock and second-order Möller – Plesset study of the lanthanide and actinide contraction. J. Chem. Phys. 109, 10806–10817. 10.1063/1.477686

[B39] Löwdin P. O. (1970). On the nonorthogonality problem. Adv. Quantum Chem. 5 (C), 185–199. 10.1016/S0065-3276(08)60339-1

[B40] Martin F. Zipse H. (2005). Charge distribution in the water molecule - a comparison of methods. J. Comput. Chem. 26 (1), 97–105. 10.1002/jcc.20157 15547940

[B41] Mayer I. (2004). Löwdin population analysis is not rotationally invariant. Chem. Phys. Lett. 393 (1–3), 209–212. 10.1016/j.cplett.2004.06.031

[B43] McLean A. D. Chandler G. S. (1980). Contracted Gaussian basis sets for molecular calculations. I. Second row atoms, Z=11-18. J. Chem. Phys. 72, 5639–5648. 10.1063/1.438980

[B44] Miliordos E. Mavridis A. (2007). Electronic structure of vanadium oxide. Neutral and charged species, VO0,±. J. Phys. Chem. A 111 (10), 1953–1965. 10.1021/jp067451b 17388278

[B45] Mulliken R. S. (1955). Electronic population analysis on LCAO–MO molecular wave functions. I. J. Chem. Phys. 23 (10), 1833–1840. 10.1063/1.1740588

[B46] Mulliken R. S. (1971). Iodine revisited. J. Chem. Phys. 55 (1), 288–309. 10.1063/1.1675521

[B47] Noell J. O. (1982). Modified electronic population analysis for transition-metal complexes. Inorg. Chem. 21, 11–14. 10.1021/ic00131a003

[B48] North S. C. Almeida N. M. S. Melin T. R. L. Wilson A. K. (2023). Multireference wavefunction-based investigation of the ground and excited states of LrF and LrO. J. Phys. Chem. A 127, 107–121. 10.1021/acs.jpca.2c06968 36596472 PMC9841984

[B49] Novir S. B. Aram M. R. (2020). Quantum mechanical simulation of Chloroquine drug interaction with C60 fullerene for treatment of COVID-19. Chem. Phys. Lett. 757, 137869. 10.1016/j.cplett.2020.137869 32834063 PMC7415227

[B50] Peterson K. A. (2015). Correlation consistent basis sets for actinides. I. the Th and U atoms. J. Chem. Phys. 142 (7), 074105. 10.1063/1.4907596 25702000

[B51] Prascher B. P. Woon D. E. Peterson K. A. Dunning T. H. Wilson A. K. (2011). Gaussian basis sets for use in correlated molecular calculations. VII. Valence, core-valence, and scalar relativistic basis sets for Li, Be, Na, and Mg. Theor. Chem. Accounts 128 (1), 69–82. 10.1007/s00214-010-0764-0

[B52] Raghavachari K. Binkley J. S. Seeger R. Pople J. A. (1980). Self-consistent molecular orbital methods. XX. A basis set for correlated wave functions. J. Chem. Phys. 72, 650–654. 10.1063/1.438955

[B53] Rangel-Galván M. Castro M. E. Perez-Aguilar J. M. Caballero N. A. Rangel-Huerta A. Melendez F. J. (2022). Theoretical study of the structural stability, chemical reactivity, and protein interaction for NMP compounds as modulators of the endocannabinoid system. Molecules 27 (2), 414. 10.3390/molecules27020414 35056729 PMC8779749

[B54] Reed A. E. Weinhold F. (1983). Natural bond orbital analysis of near-Hartree-Fock water dimer. J. Chem. Phys. 78 (6), 4066–4073. 10.1063/1.445134

[B55] Reed A. E. Weinhold F. (1985). Natural localized molecular orbitals. J. Chem. Phys. 83 (4), 1736–1740. 10.1063/1.449360

[B56] Reed A. E. Weinstock R. B. Weinhold F. (1985). Natural population analysis. J. Chem. Phys. 83 (2), 735–746. 10.1063/1.449486

[B57] Reiher M. Wolf A. (2004). Exact decoupling of the Dirac Hamiltonian. I. General theory. J. Chem. Phys. 121 (5), 2037–2047. 10.1063/1.1768160 15260757

[B58] Ritchie J. P. Bachrach S. M. (1987). Some methods and applications of electron density distribution analysis. J. Comput. Chem. 8 (4), 499–509. 10.1002/jcc.540080430

[B59] Ritchie J. P. (1985). Electron density distribution analysis for nitromethane, nitromethide, and nitramide. J. Am. Chem. Soc. 107 (7), 1829–1837. 10.1021/ja00293a005

[B60] Saha S. Roy R. K. Ayers P. W. (2009). Are the Hirshfeld and mulliken population analysis schemes consistent with chemical intuition? Int. J. Quantum Chem. 109, 1790–1806. 10.1002/qua.21901

[B61] Sigfridsson E. Ryde U. (1998). Comparison of methods for deriving atomic charges from the electrostatic potential and moments. J. Comput. Chem. 19 (4), 377–395. 10.1002/(SICI)1096-987X(199803)19:4<377:AID-JCC1>3.0.CO;2-P

[B62] Singh U. C. Kollman P. A. (1984). An approach to computing electrostatic charges for molecules. J. Comput. Chem. 5 (2), 129–145. 10.1002/jcc.540050204

[B63] Teatum E. Waber J. Gschneidner K. A. (1960). “Compilation of calculated data useful in predicting metallurgical behavior of the elements in binary alloy systems, Laboratory., Los Alamos Scientific Commission,” in U S atomic energy (United States: Los Alamos Scientific Laboratory of the University of California).

[B64] Uene N. Mabuchi T. Zaitsu M. Yasuhara S. Tokumasu T. (2022). Reactive force-field molecular dynamics simulation for the surface reaction of SiH (x = 2–4) species on Si(1 0 0)-(2 × 1):H surfaces in chemical vapor deposition processes. Comput. Mater. Sci. 204, 111193. 10.1016/j.commatsci.2022.111193

[B65] Wiberg K. B. Rablen P. R. (2018). Atomic charges. J. Org. Chem. 83 (24), 15463–15469. 10.1021/acs.joc.8b02740 30474977

[B66] Wiberg K. B. Rablen P. R. (1993). Comparison of atomic charges derived via different procedures. J. Comput. Chem. 14 (12), 1504–1518. 10.1002/jcc.540141213

[B67] Zhang Y. Elfeghe S. Tang Z. (2022). Mechanism study of Cd(II) ion adsorption onto resins with sulfonic/phosphonic groups using electronic structure methods. J. Mol. Liq. 358, 119199. 10.1016/j.molliq.2022.119199

