# Supplementary material for: Population analysis and the effects of Gaussian basis set quality and quantum mechanical approach: main group through heavy element species
Source: Front Chem. 2023 Apr 19;11:1152500. doi: 10.3389/fchem.2023.1152500 (PMC10154537; doi:10.3389/fchem.2023.1152500)

Table S1. Charge of carbon in CH_4_ and sulfur in SO_2_, using different population methods, basis sets, and quantum methods.

|  |  | **Carbon Charge in CH_4_ (ΔEN=0.45)** | | | | | | | | **Sulfur Charge in SO_2_ (ΔEN=0.86)** | | | | | | | |
| --- | --- | --- | --- | --- | --- | --- | --- | --- | --- | --- | --- | --- | --- | --- | --- | --- | --- |
|  |  | Q_LPA_ | Q_MPA_ | Q_NPA_ | Q_AIM_ | Q_HPA_ | Q_MK_ | Q_CHELP_ | Q_CHELPG_ | Q_LPA_ | Q_MPA_ | Q_NPA_ | Q_AIM_ | Q_HPA_ | Q_MK_ | Q_CHELP_ | Q_CHELPG_ |
| **B3LYP** | cc-pVDZ | -0.14 | -0.11 | -0.85 | 0.07 | -0.14 | -0.40 | 0.10 | -0.27 | 0.57 | 0.67 | 1.58 | 2.27 | 0.47 | 0.53 | 0.62 | 0.54 |
|  | cc-pVTZ | 0.09 | -0.41 | -0.81 | 0.02 | -0.13 | -0.48 | 0.06 | -0.34 | 0.24 | 0.68 | 1.60 | 2.44 | 0.45 | 0.53 | 0.65 | 0.54 |
|  | cc-pVQZ | 0.42 | -0.37 | -0.83 | 0.13 | -0.13 | -0.49 | 0.06 | -0.34 | -0.11 | 0.70 | 1.60 | 2.47 | 0.45 | 0.53 | 0.65 | 0.54 |
|  | cc-pV5Z | 0.62 | -0.18 | -0.83 | 0.08 | -0.13 | -0.49 | 0.06 | -0.34 | -0.13 | 0.71 | 1.62 | 2.46 | 0.44 | 0.53 | 0.65 | 0.54 |
|  | aug-cc-pVDZ | 0.25 | 1.26 | -0.87 | 0.10 | -0.13 | -0.49 | 0.10 | -0.32 | 0.75 | 1.10 | 1.66 | 2.33 | 0.44 | 0.52 | 0.65 | 0.54 |
|  | aug-cc-pVTZ | 0.82 | -0.93 | -0.82 | 0.02 | -0.13 | -0.48 | 0.06 | -0.33 | 0.31 | 1.12 | 1.62 | 2.46 | 0.44 | 0.52 | 0.65 | 0.54 |
|  | aug-cc-pVQZ | 1.30 | -0.98 | -0.84 | 0.13 | -0.13 | -0.48 | 0.06 | -0.34 | 0.09 | 1.37 | 1.61 | 2.47 | 0.44 | 0.52 | 0.65 | 0.54 |
|  | aug-cc-pV5Z | 1.51 | -0.72 | -0.83 | 0.08 | -0.13 | -0.48 | 0.06 | -0.33 | 0.06 | 1.04 | 1.63 | 2.46 | 0.44 | 0.52 | 0.65 | 0.54 |
|  | 6-21G** | -0.37 | -0.52 | -0.90 | -0.03 | -0.13 | -0.56 | -0.01 | -0.43 | 0.78 | 0.93 | 1.49 | 2.40 | 0.46 | 0.44 | 0.55 | 0.45 |
|  | 6-31G** | -0.46 | -0.47 | -0.93 | 0.07 | -0.14 | -0.49 | 0.01 | -0.36 | 0.94 | 0.79 | 1.60 | 2.41 | 0.48 | 0.53 | 0.64 | 0.55 |
|  | 6-311G** | -0.05 | -0.44 | -0.79 | 0.00 | -0.13 | -0.50 | 0.06 | -0.35 | 0.83 | 0.80 | 1.52 | 2.29 | 0.49 | 0.59 | 0.69 | 0.61 |
| **PBE0** | cc-pVDZ | -0.16 | -0.20 | -0.89 | 0.05 | -0.15 | -0.48 | 0.04 | -0.35 | 0.57 | 0.68 | 1.58 | 2.29 | 0.46 | 0.52 | 0.62 | 0.54 |
|  | cc-pVTZ | 0.09 | -0.43 | -0.84 | -0.04 | -0.15 | -0.55 | -0.01 | -0.41 | 0.23 | 0.65 | 1.59 | 2.48 | 0.45 | 0.52 | 0.63 | 0.53 |
|  | cc-pVQZ | 0.42 | -0.47 | -0.86 | 0.08 | -0.15 | -0.56 | 0.00 | -0.41 | -0.12 | 0.66 | 1.60 | 2.51 | 0.44 | 0.51 | 0.64 | 0.53 |
|  | cc-pV5Z | 0.63 | -0.26 | -0.86 | 0.02 | -0.15 | -0.56 | 0.00 | -0.41 | -0.14 | 0.66 | 1.62 | 2.49 | 0.44 | 0.51 | 0.64 | 0.53 |
|  | aug-cc-pVDZ | 0.23 | 0.78 | -0.89 | 0.07 | -0.14 | -0.56 | 0.03 | -0.39 | 0.75 | 1.14 | 1.66 | 2.35 | 0.43 | 0.51 | 0.64 | 0.53 |
|  | aug-cc-pVTZ | 0.82 | -0.98 | -0.85 | -0.04 | -0.15 | -0.55 | 0.00 | -0.41 | 0.30 | 1.07 | 1.62 | 2.50 | 0.43 | 0.51 | 0.64 | 0.53 |
|  | aug-cc-pVQZ | 1.30 | -1.16 | -0.87 | 0.07 | -0.15 | -0.55 | 0.00 | -0.41 | 0.08 | 1.24 | 1.61 | 2.51 | 0.43 | 0.51 | 0.64 | 0.53 |
|  | aug-cc-pV5Z | 1.52 | -0.87 | -0.86 | 0.02 | -0.15 | -0.55 | 0.00 | -0.41 | 0.05 | 0.91 | 1.62 | 2.50 | 0.44 | 0.51 | 0.64 | 0.53 |
|  | 6-21G** | -0.40 | -0.61 | -0.94 | -0.08 | -0.14 | -0.64 | -0.07 | -0.51 | 0.79 | 0.95 | 1.50 | 2.43 | 0.46 | 0.45 | 0.56 | 0.46 |
|  | 6-31G** | -0.49 | -0.57 | -0.97 | 0.03 | -0.15 | -0.57 | -0.05 | -0.44 | 0.95 | 0.81 | 1.60 | 2.44 | 0.47 | 0.53 | 0.64 | 0.55 |
|  | 6-311G** | -0.06 | -0.49 | -0.81 | -0.05 | -0.15 | -0.57 | 0.00 | -0.42 | 0.83 | 0.81 | 1.52 | 2.31 | 0.48 | 0.58 | 0.68 | 0.60 |
| **HF** | cc-pVDZ | -0.12 | -0.14 | -0.79 | 0.31 | -0.11 | -0.45 | 0.10 | -0.31 | 0.75 | 0.95 | 1.81 | 2.57 | 0.58 | 0.68 | 0.80 | 0.70 |
|  | cc-pVTZ | 0.10 | -0.37 | -0.72 | 0.15 | -0.11 | -0.50 | 0.06 | -0.35 | 0.37 | 0.86 | 1.82 | 2.81 | 0.56 | 0.65 | 0.79 | 0.66 |
|  | cc-pVQZ | 0.43 | -0.27 | -0.74 | 0.27 | -0.11 | -0.51 | 0.06 | -0.36 | -0.03 | 0.86 | 1.82 | 2.84 | 0.56 | 0.63 | 0.78 | 0.65 |
|  | cc-pV5Z | 0.64 | -0.06 | -0.74 | 0.22 | -0.11 | -0.51 | 0.06 | -0.36 | -0.08 | 0.80 | 1.84 | 2.83 | 0.56 | 0.63 | 0.78 | 0.65 |
|  | aug-cc-pVDZ | 0.24 | 0.59 | -0.78 | 0.32 | -0.11 | -0.51 | 0.09 | -0.35 | 0.89 | 1.54 | 1.88 | 2.65 | 0.55 | 0.63 | 0.78 | 0.65 |
|  | aug-cc-pVTZ | 0.83 | -1.19 | -0.72 | 0.14 | -0.11 | -0.51 | 0.06 | -0.36 | 0.37 | 1.22 | 1.84 | 2.83 | 0.55 | 0.63 | 0.78 | 0.65 |
|  | aug-cc-pVQZ | 1.31 | -0.97 | -0.75 | 0.26 | -0.11 | -0.51 | 0.06 | -0.36 | 0.10 | 1.44 | 1.83 | 2.85 | 0.55 | 0.63 | 0.78 | 0.65 |
|  | aug-cc-pV5Z | 1.52 | -0.59 | -0.74 | 0.22 | -0.11 | -0.51 | 0.06 | -0.36 | 0.06 | 1.00 | 1.85 | 2.84 | 0.55 | 0.63 | 0.78 | 0.65 |
|  | 6-21G** | -0.35 | -0.50 | -0.85 | 0.17 | -0.11 | -0.57 | 0.01 | -0.43 | 0.99 | 1.23 | 1.75 | 2.76 | 0.59 | 0.61 | 0.74 | 0.62 |
|  | 6-31G** | -0.44 | -0.47 | -0.88 | 0.24 | -0.11 | -0.50 | 0.04 | -0.36 | 1.13 | 1.09 | 1.84 | 2.74 | 0.60 | 0.70 | 0.83 | 0.71 |
|  | 6-311G** | -0.01 | -0.36 | -0.70 | 0.13 | -0.11 | -0.50 | 0.07 | -0.35 | 1.01 | 1.07 | 1.74 | 2.62 | 0.61 | 0.74 | 0.86 | 0.76 |

Table S2. Charge of carbon in CH_4_ and sulfur in SO_2_, using different population methods, basis sets, and the MP2 method.

|  | | **Carbon Charge in CH_4_ (ΔEN=0.45)** | | | | | | | | **Sulfur Charge in SO_2_ (ΔEN=0.86)** | | | | | | | |
| --- | --- | --- | --- | --- | --- | --- | --- | --- | --- | --- | --- | --- | --- | --- | --- | --- | --- |
|  |  | Q_LPA_ | Q_MPA_ | Q_NPA_ | Q_AIM_ | Q_HPA_ | Q_MK_ | Q_CHELP_ | Q_CHELPG_ | Q_LPA_ | Q_MPA_ | Q_NPA_ | Q_AIM_ | Q_HPA_ | Q_MK_ | Q_CHELP_ | Q_CHELPG_ |
| **MP2** | cc-pVDZ | -0.14 | -0.18 | -0.83 | 0.13 | -0.12 | -0.45 | 0.09 | -0.30 | 0.75 | 0.67 | 1.53 | 2.24 | 0.44 | 0.50 | 0.59 | 0.51 |
|  | cc-pVTZ | 0.08 | -0.48 | -0.79 | -0.03 | -0.13 | -0.53 | 0.03 | -0.38 | 0.58 | 0.60 | 1.54 | 2.46 | 0.42 | 0.49 | 0.60 | 0.50 |
|  | cc-pVQZ | 0.42 | -0.38 | -0.82 | 0.06 | -0.13 | -0.55 | 0.02 | -0.40 | 0.25 | 0.58 | 1.54 | 2.50 | 0.42 | 0.49 | 0.61 | 0.50 |
|  | cc-pV5Z | 0.62 | -0.18 | -0.81 | 0.01 | -0.13 | -0.55 | 0.02 | -0.40 | 0.23 | 0.51 | 1.56 | 2.50 | 0.42 | 0.49 | 0.61 | 0.50 |
|  | aug-cc-pVDZ | 0.23 | 0.67 | -0.83 | 0.13 | -0.12 | -0.52 | 0.09 | -0.35 | 1.06 | 1.17 | 1.60 | 2.33 | 0.42 | 0.49 | 0.62 | 0.50 |
|  | aug-cc-pVTZ | 0.81 | -1.25 | -0.80 | -0.04 | -0.13 | -0.54 | 0.02 | -0.39 | 0.85 | 0.91 | 1.56 | 2.49 | 0.41 | 0.48 | 0.61 | 0.50 |
|  | aug-cc-pVQZ | 1.29 | -0.98 | -0.82 | 0.05 | -0.13 | -0.55 | 0.02 | -0.40 | 0.56 | 1.14 | 1.56 | 2.51 | 0.42 | 0.48 | 0.61 | 0.50 |
|  | aug-cc-pV5Z | 1.51 | -0.68 | -0.82 | 0.01 | -0.13 | -0.55 | 0.02 | -0.40 | 0.50 | 0.75 | 1.57 | 2.50 | 0.42 | 0.48 | 0.61 | 0.50 |
|  | 6-21G** | -0.35 | -0.50 | -0.87 | 0.00 | -0.11 | -0.55 | 0.01 | -0.42 | 0.76 | 0.93 | 1.44 | 2.41 | 0.43 | 0.42 | 0.53 | 0.44 |
|  | 6-31G** | -0.45 | -0.47 | -0.90 | 0.10 | -0.12 | -0.48 | 0.04 | -0.35 | 0.93 | 0.80 | 1.55 | 2.41 | 0.45 | 0.52 | 0.63 | 0.54 |
|  | 6-311G** | -0.04 | -0.42 | -0.75 | -0.01 | -0.12 | -0.50 | 0.06 | -0.35 | 0.80 | 0.78 | 1.46 | 2.27 | 0.46 | 0.56 | 0.65 | 0.57 |

Table S3. Charge of carbon in CO_2_ and nitrogen in NH_3_, using different population methods, basis sets, and quantum methods.

|  |  | |  | **Carbon Charge in CO_2_ (ΔEN=0.89)** | | | | | | | | **Nitrogen Charge in NH_3_ (ΔEN=0.94)** | | | | | | | |
| --- | --- | --- | --- | --- | --- | --- | --- | --- | --- | --- | --- | --- | --- | --- | --- | --- | --- | --- | --- |
|  |  | |  | Q_LPA_ | Q_MPA_ | Q_NPA_ | Q_AIM_ | Q_HPA_ | Q_MK_ | Q_CHELP_ | Q_CHELPG_ | Q_LPA_ | Q_MPA_ | Q_NPA_ | Q_AIM_ | Q_HPA_ | Q_MK_ | Q_CHELP_ | Q_CHELPG_ |
| **B3LYP** |  | cc-pVDZ | | -0.16 | 0.28 | 1.05 | 2.30 | 0.35 | 0.67 | 0.60 | 0.71 | -0.13 | -0.21 | -1.05 | -1.20 | -0.30 | -0.89 | -0.86 | -0.89 |
|  | cc-pVTZ | | | -0.52 | 0.37 | 0.98 | 2.24 | 0.35 | 0.71 | 0.61 | 0.76 | 0.28 | -0.46 | -1.03 | -1.00 | -0.29 | -0.89 | -0.86 | -0.89 |
|  | cc-pVQZ | | | -0.88 | 0.58 | 0.99 | 2.30 | 0.35 | 0.71 | 0.61 | 0.77 | 0.75 | -0.51 | -1.06 | -1.05 | -0.28 | -0.88 | -0.85 | -0.88 |
|  | cc-pV5Z | | | -1.10 | 0.67 | 0.98 | 2.28 | 0.35 | 0.72 | 0.62 | 0.77 | 1.00 | -0.66 | -1.05 | -1.03 | -0.28 | -0.88 | -0.83 | -0.87 |
|  | aug-cc-pVDZ | | | -0.37 | 0.35 | 1.07 | 2.29 | 0.34 | 0.73 | 0.62 | 0.78 | 0.28 | 0.16 | -1.12 | -1.17 | -0.27 | -0.87 | -0.82 | -0.86 |
|  | aug-cc-pVTZ | | | -0.72 | 0.38 | 0.99 | 2.25 | 0.35 | 0.72 | 0.62 | 0.78 | 1.05 | -0.44 | -1.05 | -1.01 | -0.27 | -0.87 | -0.83 | -0.86 |
|  | aug-cc-pVQZ | | | -1.13 | 0.88 | 0.98 | 2.30 | 0.35 | 0.72 | 0.61 | 0.77 | 1.61 | -0.68 | -1.07 | -1.05 | -0.28 | -0.87 | -0.83 | -0.86 |
|  | aug-cc-pV5Z | | | -1.27 | 0.79 | 0.98 | 2.28 | 0.35 | 0.72 | 0.61 | 0.77 | 1.83 | -1.08 | -1.05 | -1.03 | -0.28 | -0.87 | -0.83 | -0.86 |
|  | 6-21G** | | | 0.13 | 0.79 | 0.94 | 2.22 | 0.34 | 0.60 | 0.55 | 0.64 | -0.37 | -0.50 | -1.04 | -0.99 | -0.29 | -0.89 | -0.88 | -0.90 |
|  | 6-31G** | | | 0.23 | 0.72 | 1.02 | 2.32 | 0.36 | 0.71 | 0.62 | 0.75 | -0.52 | -0.72 | -1.13 | -1.05 | -0.30 | -1.01 | -0.97 | -1.00 |
|  | 6-311G** | | | -0.06 | 0.50 | 1.00 | 2.13 | 0.35 | 0.74 | 0.65 | 0.78 | -0.07 | -0.57 | -1.01 | -0.96 | -0.29 | -0.96 | -0.92 | -0.96 |
| **PBE0** | cc-pVDZ | | | -0.15 | 0.32 | 1.06 | 2.32 | 0.35 | 0.67 | 0.60 | 0.71 | -0.13 | -0.25 | -1.07 | -1.22 | -0.30 | -0.91 | -0.88 | -0.91 |
|  | cc-pVTZ | | | -0.52 | 0.36 | 0.98 | 2.29 | 0.35 | 0.69 | 0.59 | 0.74 | 0.28 | -0.45 | -1.04 | -1.04 | -0.29 | -0.90 | -0.87 | -0.90 |
|  | cc-pVQZ | | | -0.88 | 0.63 | 0.99 | 2.34 | 0.35 | 0.69 | 0.59 | 0.75 | 0.75 | -0.54 | -1.07 | -1.09 | -0.29 | -0.89 | -0.85 | -0.88 |
|  | cc-pV5Z | | | -1.11 | 0.66 | 0.98 | 2.33 | 0.35 | 0.70 | 0.60 | 0.75 | 1.01 | -0.70 | -1.06 | -1.08 | -0.28 | -0.88 | -0.84 | -0.87 |
|  | aug-cc-pVDZ | | | -0.36 | 0.45 | 1.07 | 2.32 | 0.34 | 0.71 | 0.61 | 0.76 | 0.27 | -0.01 | -1.13 | -1.20 | -0.28 | -0.88 | -0.83 | -0.87 |
|  | aug-cc-pVTZ | | | -0.72 | 0.33 | 0.99 | 2.30 | 0.35 | 0.70 | 0.60 | 0.75 | 1.05 | -0.44 | -1.06 | -1.06 | -0.28 | -0.87 | -0.83 | -0.86 |
|  | aug-cc-pVQZ | | | -1.14 | 0.97 | 0.99 | 2.35 | 0.35 | 0.69 | 0.60 | 0.75 | 1.62 | -0.72 | -1.08 | -1.10 | -0.28 | -0.87 | -0.83 | -0.87 |
|  | aug-cc-pV5Z | | | -1.28 | 0.81 | 0.98 | 2.33 | 0.35 | 0.69 | 0.60 | 0.75 | 1.84 | -1.18 | -1.06 | -1.08 | -0.28 | -0.87 | -0.83 | -0.87 |
|  | 6-21G** | | | 0.14 | 0.81 | 0.96 | 2.26 | 0.34 | 0.60 | 0.55 | 0.64 | -0.38 | -0.55 | -1.06 | -1.02 | -0.30 | -0.92 | -0.90 | -0.92 |
|  | 6-31G** | | | 0.23 | 0.73 | 1.03 | 2.36 | 0.35 | 0.70 | 0.61 | 0.74 | -0.53 | -0.77 | -1.15 | -1.09 | -0.31 | -1.03 | -0.99 | -1.03 |
|  | 6-311G** | | | -0.05 | 0.52 | 1.00 | 2.17 | 0.35 | 0.73 | 0.64 | 0.77 | -0.08 | -0.59 | -1.02 | -1.00 | -0.30 | -0.97 | -0.94 | -0.97 |
| **HF** | cc-pVDZ | | | -0.01 | 0.52 | 1.29 | 2.64 | 0.45 | 0.85 | 0.76 | 0.90 | -0.13 | -0.27 | -1.05 | -1.24 | -0.30 | -0.95 | -0.92 | -0.95 |
|  | cc-pVTZ | | | -0.42 | 0.54 | 1.21 | 2.68 | 0.45 | 0.83 | 0.72 | 0.88 | 0.28 | -0.49 | -1.01 | -1.09 | -0.29 | -0.93 | -0.90 | -0.93 |
|  | cc-pVQZ | | | -0.82 | 0.72 | 1.22 | 2.73 | 0.45 | 0.82 | 0.72 | 0.89 | 0.76 | -0.52 | -1.04 | -1.14 | -0.29 | -0.93 | -0.89 | -0.92 |
|  | cc-pV5Z | | | -1.06 | 0.72 | 1.21 | 2.71 | 0.45 | 0.83 | 0.72 | 0.89 | 1.02 | -0.58 | -1.03 | -1.12 | -0.29 | -0.92 | -0.89 | -0.92 |
|  | aug-cc-pVDZ | | | -0.26 | 0.61 | 1.30 | 2.66 | 0.44 | 0.84 | 0.73 | 0.90 | 0.26 | -0.10 | -1.10 | -1.23 | -0.28 | -0.92 | -0.88 | -0.92 |
|  | aug-cc-pVTZ | | | -0.70 | 0.49 | 1.21 | 2.69 | 0.45 | 0.83 | 0.72 | 0.89 | 1.06 | -0.60 | -1.03 | -1.11 | -0.28 | -0.92 | -0.88 | -0.91 |
|  | aug-cc-pVQZ | | | -1.12 | 1.00 | 1.21 | 2.73 | 0.45 | 0.82 | 0.72 | 0.89 | 1.63 | -0.73 | -1.05 | -1.15 | -0.28 | -0.92 | -0.88 | -0.91 |
|  | aug-cc-pV5Z | | | -1.28 | 0.79 | 1.21 | 2.71 | 0.45 | 0.82 | 0.72 | 0.89 | 1.85 | -0.98 | -1.04 | -1.12 | -0.28 | -0.92 | -0.88 | -0.91 |
|  | 6-21G** | | | 0.30 | 0.98 | 1.20 | 2.63 | 0.45 | 0.78 | 0.72 | 0.83 | -0.38 | -0.56 | -1.05 | -1.04 | -0.30 | -0.95 | -0.93 | -0.95 |
|  | 6-31G** | | | 0.38 | 0.91 | 1.26 | 2.71 | 0.46 | 0.88 | 0.77 | 0.93 | -0.53 | -0.79 | -1.13 | -1.10 | -0.30 | -1.06 | -1.02 | -1.05 |
|  | 6-311G** | | | 0.09 | 0.72 | 1.22 | 2.53 | 0.46 | 0.90 | 0.79 | 0.94 | -0.07 | -0.56 | -0.99 | -1.04 | -0.30 | -0.99 | -0.96 | -0.99 |

Table S4. Charge of carbon in CO_2_ and nitrogen in NH_3_, using different population methods, basis sets, and the MP2 method.

|  | | **Carbon Charge in CO_2_ (ΔEN=0.89)** | | | | | | | | **Nitrogen Charge in NH_3_ (ΔEN=0.94)** | | | | | | | |
| --- | --- | --- | --- | --- | --- | --- | --- | --- | --- | --- | --- | --- | --- | --- | --- | --- | --- |
|  |  | Q_LPA_ | Q_MPA_ | Q_NPA_ | Q_AIM_ | Q_HPA_ | Q_MK_ | Q_CHELP_ | Q_CHELPG_ | Q_LPA_ | Q_MPA_ | Q_NPA_ | Q_AIM_ | Q_HPA_ | Q_MK_ | Q_CHELP_ | Q_CHELPG_ |
| **MP2** | cc-pVDZ | -0.18 | 0.28 | 1.01 | 2.29 | 0.31 | 0.66 | 0.58 | 0.70 | -0.13 | -0.26 | -1.04 | -1.21 | -0.26 | -0.92 | -0.89 | -0.92 |
|  | cc-pVTZ | -0.54 | 0.33 | 0.93 | 2.30 | 0.31 | 0.66 | 0.57 | 0.71 | 0.28 | -0.53 | -1.03 | -1.09 | -0.27 | -0.90 | -0.87 | -0.90 |
|  | cc-pVQZ | -0.90 | 0.50 | 0.93 | 2.35 | 0.31 | 0.66 | 0.56 | 0.71 | 0.75 | -0.53 | -1.06 | -1.15 | -0.27 | -0.89 | -0.86 | -0.89 |
|  | cc-pV5Z | -1.11 | 0.51 | 0.92 | 2.35 | 0.31 | 0.66 | 0.57 | 0.72 | 1.00 | -0.60 | -1.06 | -1.13 | -0.27 | -0.89 | -0.85 | -0.88 |
|  | aug-cc-pVDZ | -0.37 | 0.35 | 1.03 | 2.31 | 0.30 | 0.69 | 0.59 | 0.75 | 0.27 | -0.03 | -1.11 | -1.22 | -0.28 | -0.88 | -0.83 | -0.87 |
|  | aug-cc-pVTZ | -0.72 | 0.34 | 0.94 | 2.31 | 0.31 | 0.67 | 0.57 | 0.72 | 1.04 | -0.60 | -1.05 | -1.11 | -0.28 | -0.88 | -0.83 | -0.87 |
|  | aug-cc-pVQZ | -1.13 | 0.78 | 0.93 | 2.36 | 0.31 | 0.66 | 0.56 | 0.72 | 1.61 | -0.68 | -1.06 | -1.16 | -0.28 | -0.88 | -0.84 | -0.87 |
|  | aug-cc-pV5Z | -1.28 | 0.62 | 0.92 | 2.35 | 0.31 | 0.66 | 0.56 | 0.72 | 1.83 | -0.97 | -1.05 | -1.13 | -0.28 | -0.88 | -0.84 | -0.87 |
|  | 6-21G** | 0.11 | 0.72 | 0.90 | 2.25 | 0.30 | 0.59 | 0.54 | 0.62 | -0.37 | -0.52 | -1.03 | -1.00 | -0.28 | -0.92 | -0.90 | -0.92 |
|  | 6-31G** | 0.21 | 0.65 | 0.98 | 2.34 | 0.31 | 0.69 | 0.60 | 0.74 | -0.52 | -0.75 | -1.12 | -1.06 | -0.28 | -1.03 | -0.99 | -1.03 |
|  | 6-311G** | -0.09 | 0.48 | 0.93 | 2.15 | 0.31 | 0.70 | 0.62 | 0.74 | -0.07 | -0.56 | -0.99 | -1.01 | -0.28 | -0.97 | -0.93 | -0.97 |

Table S5. Charge of oxygen in H_2_O and nitrogen in NH_3_, using different population methods, basis sets, and quantum methods.

|  |  | **Oxygen Charge in H_2_O (ΔEN=1.34)** | | | | | | | | **Beryllium Charge in BeCl_2_ (ΔEN=1.59)** | | | | | | | |
| --- | --- | --- | --- | --- | --- | --- | --- | --- | --- | --- | --- | --- | --- | --- | --- | --- | --- |
|  |  | Q_LPA_ | Q_MPA_ | Q_NPA_ | Q_AIM_ | Q_HPA_ | Q_MK_ | Q_CHELP_ | Q_CHELPG_ | Q_LPA_ | Q_MPA_ | Q_NPA_ | Q_AIM_ | Q_HPA_ | Q_MK_ | Q_CHELP_ | Q_CHELPG_ |
| **B3LYP** | cc-pVDZ | -0.09 | -0.26 | -0.89 | -1.18 | -0.33 | -0.70 | -0.70 | -0.69 | -0.68 | 0.06 | 0.83 | 1.71 | 0.40 | 0.36 | 0.33 | 0.45 |
|  | cc-pVTZ | 0.35 | -0.43 | -0.90 | -1.13 | -0.31 | -0.70 | -0.70 | -0.70 | -1.26 | 0.17 | 0.79 | 1.68 | 0.40 | 0.43 | 0.40 | 0.52 |
|  | cc-pVQZ | 0.85 | -0.49 | -0.92 | -1.14 | -0.31 | -0.70 | -0.70 | -0.69 | -1.63 | 0.54 | 0.80 | 1.69 | 0.41 | 0.43 | 0.41 | 0.52 |
|  | cc-pV5Z | 1.13 | -0.56 | -0.91 | -1.13 | -0.30 | -0.69 | -0.69 | -0.69 | -2.09 | 0.64 | 0.78 | 1.68 | 0.40 | 0.43 | 0.41 | 0.52 |
|  | aug-cc-pVDZ | 0.22 | -0.15 | -0.95 | -1.17 | -0.29 | -0.68 | -0.68 | -0.68 | -0.77 | -0.27 | 0.86 | 1.69 | 0.40 | 0.43 | 0.40 | 0.51 |
|  | aug-cc-pVTZ | 1.01 | -0.35 | -0.92 | -1.15 | -0.30 | -0.68 | -0.68 | -0.68 | -1.61 | 0.07 | 0.79 | 1.68 | 0.40 | 0.43 | 0.41 | 0.52 |
|  | aug-cc-pVQZ | 1.59 | -0.58 | -0.92 | -1.14 | -0.30 | -0.68 | -0.68 | -0.68 | -2.05 | 0.98 | 0.79 | 1.68 | 0.40 | 0.43 | 0.41 | 0.52 |
|  | aug-cc-pV5Z | 1.84 | -0.85 | -0.91 | -1.13 | -0.30 | -0.68 | -0.68 | -0.68 | -2.52 | 1.35 | 0.78 | 1.68 | 0.40 | 0.43 | 0.41 | 0.52 |
|  | 6-21G** | -0.30 | -0.47 | -0.85 | -1.00 | -0.32 | -0.65 | -0.65 | -0.65 | -0.06 | 0.51 | 0.81 | 1.67 | 0.41 | 0.48 | 0.45 | 0.56 |
|  | 6-31G** | -0.43 | -0.61 | -0.94 | -1.14 | -0.33 | -0.74 | -0.74 | -0.74 | -0.33 | 0.30 | 0.82 | 1.69 | 0.41 | 0.45 | 0.41 | 0.52 |
|  | 6-311G** | -0.06 | -0.48 | -0.88 | -1.07 | -0.33 | -0.75 | -0.75 | -0.75 | -0.66 | 0.30 | 0.79 | 1.65 | 0.39 | 0.46 | 0.43 | 0.55 |
| **PBE0** | cc-pVDZ | -0.09 | -0.27 | -0.90 | -1.20 | -0.33 | -0.70 | -0.71 | -0.70 | -0.68 | 0.07 | 0.84 | 1.71 | 0.40 | 0.36 | 0.33 | 0.45 |
|  | cc-pVTZ | 0.35 | -0.43 | -0.90 | -1.16 | -0.32 | -0.70 | -0.71 | -0.70 | -1.26 | 0.17 | 0.79 | 1.69 | 0.41 | 0.42 | 0.39 | 0.51 |
|  | cc-pVQZ | 0.85 | -0.49 | -0.92 | -1.16 | -0.31 | -0.70 | -0.70 | -0.69 | -1.64 | 0.58 | 0.80 | 1.70 | 0.41 | 0.42 | 0.40 | 0.51 |
|  | cc-pV5Z | 1.14 | -0.58 | -0.91 | -1.15 | -0.31 | -0.70 | -0.69 | -0.69 | -2.10 | 0.62 | 0.79 | 1.69 | 0.41 | 0.43 | 0.40 | 0.51 |
|  | aug-cc-pVDZ | 0.21 | -0.22 | -0.96 | -1.19 | -0.30 | -0.69 | -0.68 | -0.68 | -0.76 | -0.18 | 0.85 | 1.70 | 0.40 | 0.42 | 0.38 | 0.50 |
|  | aug-cc-pVTZ | 1.01 | -0.35 | -0.92 | -1.18 | -0.30 | -0.68 | -0.68 | -0.68 | -1.61 | 0.07 | 0.79 | 1.69 | 0.41 | 0.42 | 0.40 | 0.51 |
|  | aug-cc-pVQZ | 1.60 | -0.59 | -0.92 | -1.17 | -0.30 | -0.68 | -0.68 | -0.68 | -2.06 | 1.09 | 0.79 | 1.70 | 0.41 | 0.43 | 0.40 | 0.51 |
|  | aug-cc-pV5Z | 1.85 | -0.92 | -0.91 | -1.16 | -0.30 | -0.68 | -0.68 | -0.68 | -2.53 | 1.32 | 0.78 | 1.69 | 0.41 | 0.43 | 0.40 | 0.51 |
|  | 6-21G** | -0.31 | -0.50 | -0.86 | -1.03 | -0.33 | -0.67 | -0.67 | -0.67 | -0.05 | 0.54 | 0.82 | 1.67 | 0.42 | 0.50 | 0.46 | 0.57 |
|  | 6-31G** | -0.44 | -0.63 | -0.95 | -1.16 | -0.33 | -0.75 | -0.76 | -0.75 | -0.33 | 0.32 | 0.82 | 1.70 | 0.41 | 0.45 | 0.42 | 0.53 |
|  | 6-311G** | -0.06 | -0.48 | -0.88 | -1.10 | -0.33 | -0.76 | -0.76 | -0.75 | -0.67 | 0.30 | 0.79 | 1.66 | 0.40 | 0.45 | 0.42 | 0.53 |
| **HF** | cc-pVDZ | -0.11 | -0.31 | -0.91 | -1.26 | -0.34 | -0.75 | -0.75 | -0.75 | -0.61 | 0.18 | 0.95 | 1.75 | 0.45 | 0.48 | 0.44 | 0.56 |
|  | cc-pVTZ | 0.34 | -0.48 | -0.91 | -1.25 | -0.33 | -0.74 | -0.74 | -0.74 | -1.22 | 0.28 | 0.89 | 1.75 | 0.46 | 0.51 | 0.48 | 0.60 |
|  | cc-pVQZ | 0.85 | -0.53 | -0.92 | -1.24 | -0.33 | -0.74 | -0.74 | -0.73 | -1.61 | 0.61 | 0.90 | 1.76 | 0.46 | 0.52 | 0.49 | 0.60 |
|  | cc-pV5Z | 1.14 | -0.56 | -0.91 | -1.24 | -0.33 | -0.73 | -0.73 | -0.73 | -2.08 | 0.59 | 0.89 | 1.76 | 0.46 | 0.52 | 0.49 | 0.60 |
|  | aug-cc-pVDZ | 0.18 | -0.30 | -0.96 | -1.25 | -0.32 | -0.73 | -0.73 | -0.73 | -0.72 | -0.08 | 0.96 | 1.75 | 0.45 | 0.51 | 0.48 | 0.59 |
|  | aug-cc-pVTZ | 1.00 | -0.44 | -0.92 | -1.27 | -0.32 | -0.73 | -0.73 | -0.72 | -1.61 | 0.20 | 0.89 | 1.75 | 0.46 | 0.51 | 0.48 | 0.60 |
|  | aug-cc-pVQZ | 1.60 | -0.58 | -0.92 | -1.24 | -0.32 | -0.73 | -0.72 | -0.72 | -2.06 | 1.10 | 0.89 | 1.76 | 0.46 | 0.51 | 0.48 | 0.60 |
|  | aug-cc-pV5Z | 1.85 | -0.80 | -0.91 | -1.24 | -0.32 | -0.73 | -0.73 | -0.72 | -2.53 | 1.05 | 0.88 | 1.76 | 0.46 | 0.51 | 0.48 | 0.60 |
|  | 6-21G** | -0.33 | -0.54 | -0.88 | -1.10 | -0.34 | -0.71 | -0.71 | -0.71 | 0.03 | 0.68 | 0.93 | 1.74 | 0.47 | 0.63 | 0.59 | 0.70 |
|  | 6-31G** | -0.45 | -0.67 | -0.97 | -1.22 | -0.35 | -0.79 | -0.80 | -0.79 | -0.26 | 0.46 | 0.93 | 1.75 | 0.46 | 0.57 | 0.54 | 0.65 |
|  | 6-311G** | -0.07 | -0.50 | -0.89 | -1.18 | -0.34 | -0.79 | -0.79 | -0.79 | -0.61 | 0.37 | 0.89 | 1.72 | 0.45 | 0.55 | 0.52 | 0.64 |

Table S6. Charge of oxygen in H_2_O and nitrogen in NH_3_, using different population methods, basis sets, and the MP2 method.

|  | | **Oxygen Charge in H_2_O (ΔEN=1.34)** | | | | | | | | **Beryllium Charge in BeCl_2_ (ΔEN=1.59)** | | | | | | | |
| --- | --- | --- | --- | --- | --- | --- | --- | --- | --- | --- | --- | --- | --- | --- | --- | --- | --- |
|  |  | Q_LPA_ | Q_MPA_ | Q_NPA_ | Q_AIM_ | Q_HPA_ | Q_MK_ | Q_CHELP_ | Q_CHELPG_ | Q_LPA_ | Q_MPA_ | Q_NPA_ | Q_AIM_ | Q_HPA_ | Q_MK_ | Q_CHELP_ | Q_CHELPG_ |
| **MP2** | cc-pVDZ | -0.02 | -0.23 | -0.88 | -1.30 | -0.32 | -0.80 | -0.81 | -0.80 | -0.50 | 0.17 | 1.40 | 1.72 | 0.35 | 0.43 | 0.37 | 0.69 |
|  | cc-pVTZ | 0.52 | -0.46 | -0.91 | -1.31 | -0.31 | -0.78 | -0.80 | -0.79 | -1.16 | 0.29 | 1.42 | 1.73 | 0.36 | 0.46 | 0.41 | 0.69 |
|  | cc-pVQZ | 1.07 | -0.45 | -0.92 | -1.31 | -0.31 | -0.78 | -0.79 | -0.78 | -1.58 | 0.58 | 1.43 | 1.74 | 0.37 | 0.47 | 0.42 | 0.69 |
|  | cc-pV5Z | 1.36 | -0.52 | -0.92 | -1.32 | -0.30 | -0.77 | -0.78 | -0.77 | -2.11 | 0.53 | 1.43 | 1.74 | 0.37 | 0.47 | 0.42 | 0.69 |
|  | aug-cc-pVDZ | 0.32 | -0.10 | -0.95 | -1.27 | -0.29 | -0.76 | -0.77 | -0.77 | -0.62 | 0.07 | 1.45 | 1.72 | 0.35 | 0.48 | 0.42 | 0.64 |
|  | aug-cc-pVTZ | 1.15 | -0.31 | -0.93 | -1.33 | -0.29 | -0.76 | -0.76 | -0.76 | -1.75 | 0.15 | 1.44 | 1.73 | 0.36 | 0.47 | 0.41 | 0.68 |
|  | aug-cc-pVQZ | 1.74 | -0.44 | -0.93 | -1.32 | -0.30 | -0.76 | -0.77 | -0.76 | -2.43 | 0.90 | 1.43 | 1.74 | 0.37 | 0.47 | 0.42 | 0.69 |
|  | aug-cc-pV5Z | 1.98 | -0.80 | -0.92 | -1.32 | -0.30 | -0.76 | -0.77 | -0.76 | -2.85 | 1.02 | 1.43 | 1.74 | 0.37 | 0.47 | 0.42 | 0.69 |
|  | 6-21G** | -0.27 | -0.46 | -0.87 | - | -0.33 | -0.76 | -0.77 | -0.76 | 0.05 | 0.61 | 1.37 | 1.69 | 0.37 | 0.56 | 0.50 | 0.75 |
|  | 6-31G** | -0.39 | -0.64 | -0.94 | -1.25 | -0.32 | -0.85 | -0.86 | -0.85 | -0.21 | 0.41 | 1.39 | 1.72 | 0.36 | 0.51 | 0.45 | 0.70 |
|  | 6-311G** | 0.02 | -0.51 | -0.88 | -1.22 | -0.32 | -0.85 | -0.86 | -0.85 | -0.55 | 0.34 | 1.39 | 1.69 | 0.34 | 0.49 | 0.43 | 0.69 |

Table S7. Charge of magnesium in MgCl_2_ and fluorine in HF, using different population methods, basis sets, and quantum methods.

|  |  | **Magnesium Charge in MgCl_2_ (ΔEN=1.85)** | | | | | | | | **Fluorine Charge in HF (ΔEN=1.88)** | | | | | | | |
| --- | --- | --- | --- | --- | --- | --- | --- | --- | --- | --- | --- | --- | --- | --- | --- | --- | --- |
|  |  | Q_LPA_ | Q_MPA_ | Q_NPA_ | Q_AIM_ | Q_HPA_ | Q_MK_ | Q_CHELP_ | Q_CHELPG_ | Q_LPA_ | Q_MPA_ | Q_NPA_ | Q_AIM_ | Q_HPA_ | Q_MK_ | Q_CHELP_ | Q_CHELPG_ |
| **B3LYP** | cc-pVDZ | 0.12 | 0.64 | 1.33 | 1.67 | 0.75 | 0.92 | 0.84 | 0.86 | -0.06 | -0.21 | -0.52 | -0.72 | -0.23 | -0.41 | -0.40 | -0.41 |
|  | cc-pVTZ | -0.37 | 0.71 | 1.39 | 1.68 | 0.75 | 0.94 | 0.86 | 0.87 | 0.25 | -0.32 | -0.54 | -0.72 | -0.22 | -0.42 | -0.40 | -0.42 |
|  | cc-pVQZ | -0.93 | 0.74 | 1.41 | 1.67 | 0.74 | 0.94 | 0.86 | 0.87 | 0.62 | -0.34 | -0.55 | -0.72 | -0.21 | -0.42 | -0.40 | -0.42 |
|  | cc-pV5Z | -1.31 | 0.73 | 1.39 | 1.67 | 0.74 | 0.95 | 0.87 | 0.87 | 0.85 | -0.37 | -0.54 | -0.73 | -0.21 | -0.42 | -0.40 | -0.42 |
|  | aug-cc-pVDZ | 0.18 | 0.59 | 1.42 | 1.69 | 0.74 | 0.93 | 0.84 | 0.85 | 0.09 | -0.25 | -0.56 | -0.71 | -0.21 | -0.41 | -0.39 | -0.41 |
|  | aug-cc-pVTZ | -0.67 | 0.73 | 1.41 | 1.68 | 0.74 | 0.95 | 0.87 | 0.87 | 0.66 | -0.33 | -0.55 | -0.73 | -0.21 | -0.41 | -0.40 | -0.41 |
|  | aug-cc-pVQZ | -1.42 | 1.00 | 1.40 | 1.67 | 0.74 | 0.95 | 0.87 | 0.87 | 1.13 | -0.43 | -0.55 | -0.73 | -0.21 | -0.41 | -0.40 | -0.41 |
|  | aug-cc-pV5Z | -1.93 | 0.96 | 1.39 | 1.67 | 0.74 | 0.95 | 0.87 | 0.87 | 1.35 | -0.52 | -0.54 | -0.73 | -0.21 | -0.41 | -0.40 | -0.41 |
|  | 6-21G** | 0.05 | 0.59 | 1.31 | 1.67 | 0.75 | 0.93 | 0.85 | 0.86 | -0.18 | -0.32 | -0.48 | -0.65 | -0.23 | -0.37 | -0.36 | -0.37 |
|  | 6-31G** | 0.15 | 0.53 | 1.34 | 1.66 | 0.74 | 0.94 | 0.86 | 0.87 | -0.25 | -0.36 | -0.54 | -0.70 | -0.23 | -0.42 | -0.40 | -0.42 |
|  | 6-311G** | 0.10 | 0.80 | 1.39 | 1.65 | 0.72 | 0.99 | 0.92 | 0.92 | -0.04 | -0.30 | -0.53 | -0.69 | -0.23 | -0.44 | -0.42 | -0.44 |
| **PBE0** | cc-pVDZ | 0.13 | 0.67 | 1.35 | 1.69 | 0.77 | 0.94 | 0.86 | 0.88 | -0.06 | -0.21 | -0.53 | -0.73 | -0.23 | -0.42 | -0.40 | -0.42 |
|  | cc-pVTZ | -0.37 | 0.72 | 1.40 | 1.70 | 0.77 | 0.95 | 0.87 | 0.87 | 0.25 | -0.32 | -0.54 | -0.73 | -0.22 | -0.42 | -0.41 | -0.42 |
|  | cc-pVQZ | -0.92 | 0.78 | 1.42 | 1.69 | 0.76 | 0.95 | 0.87 | 0.88 | 0.62 | -0.34 | -0.55 | -0.74 | -0.22 | -0.42 | -0.40 | -0.42 |
|  | cc-pV5Z | -1.31 | 0.78 | 1.42 | 1.69 | 0.76 | 0.95 | 0.87 | 0.88 | 0.85 | -0.37 | -0.54 | -0.74 | -0.21 | -0.42 | -0.40 | -0.42 |
|  | aug-cc-pVDZ | 0.19 | 0.73 | 1.43 | 1.71 | 0.76 | 0.94 | 0.85 | 0.86 | 0.08 | -0.28 | -0.56 | -0.72 | -0.21 | -0.41 | -0.40 | -0.41 |
|  | aug-cc-pVTZ | -0.66 | 0.78 | 1.42 | 1.70 | 0.76 | 0.95 | 0.87 | 0.88 | 0.66 | -0.35 | -0.55 | -0.74 | -0.21 | -0.42 | -0.40 | -0.41 |
|  | aug-cc-pVQZ | -1.42 | 1.06 | 1.42 | 1.69 | 0.76 | 0.95 | 0.87 | 0.88 | 1.13 | -0.43 | -0.55 | -0.74 | -0.21 | -0.42 | -0.40 | -0.41 |
|  | aug-cc-pV5Z | -1.93 | 1.06 | 1.41 | 1.69 | 0.76 | 0.95 | 0.87 | 0.88 | 1.35 | -0.54 | -0.54 | -0.74 | -0.21 | -0.41 | -0.40 | -0.41 |
|  | 6-21G** | 0.06 | 0.60 | 1.33 | 1.69 | 0.76 | 0.95 | 0.87 | 0.88 | -0.19 | -0.34 | -0.49 | -0.67 | -0.23 | -0.38 | -0.37 | -0.38 |
|  | 6-31G** | 0.16 | 0.54 | 1.36 | 1.68 | 0.76 | 0.96 | 0.88 | 0.89 | -0.26 | -0.36 | -0.55 | -0.71 | -0.24 | -0.42 | -0.41 | -0.42 |
|  | 6-311G** | 0.11 | 0.81 | 1.40 | 1.66 | 0.74 | 1.00 | 0.92 | 0.93 | -0.04 | -0.30 | -0.53 | -0.70 | -0.23 | -0.44 | -0.42 | -0.44 |
| **HF** | cc-pVDZ | 0.21 | 0.83 | 1.45 | 1.74 | 0.83 | 1.06 | 0.98 | 0.99 | -0.07 | -0.24 | -0.55 | -0.77 | -0.25 | -0.45 | -0.43 | -0.45 |
|  | cc-pVTZ | -0.31 | 0.85 | 1.49 | 1.76 | 0.83 | 1.04 | 0.96 | 0.97 | 0.23 | -0.35 | -0.55 | -0.78 | -0.24 | -0.45 | -0.43 | -0.45 |
|  | cc-pVQZ | -0.88 | 0.99 | 1.49 | 1.76 | 0.82 | 1.04 | 0.96 | 0.97 | 0.61 | -0.37 | -0.55 | -0.79 | -0.24 | -0.44 | -0.43 | -0.44 |
|  | cc-pV5Z | -1.28 | 0.95 | 1.49 | 1.76 | 0.82 | 1.04 | 0.96 | 0.97 | 0.85 | -0.39 | -0.55 | -0.79 | -0.24 | -0.44 | -0.43 | -0.44 |
|  | aug-cc-pVDZ | 0.23 | 1.15 | 1.51 | 1.76 | 0.82 | 1.03 | 0.94 | 0.96 | 0.05 | -0.32 | -0.57 | -0.76 | -0.23 | -0.44 | -0.43 | -0.44 |
|  | aug-cc-pVTZ | -0.65 | 0.89 | 1.50 | 1.76 | 0.82 | 1.04 | 0.96 | 0.97 | 0.64 | -0.37 | -0.56 | -0.78 | -0.23 | -0.44 | -0.43 | -0.44 |
|  | aug-cc-pVQZ | -1.42 | 1.57 | 1.49 | 1.76 | 0.82 | 1.04 | 0.96 | 0.97 | 1.12 | -0.43 | -0.55 | -0.79 | -0.23 | -0.44 | -0.43 | -0.44 |
|  | aug-cc-pV5Z | -1.95 | 1.29 | 1.49 | 1.76 | 0.82 | 1.04 | 0.96 | 0.97 | 1.35 | -0.52 | -0.55 | -0.79 | -0.23 | -0.44 | -0.43 | -0.44 |
|  | 6-21G** | 0.14 | 0.72 | 1.43 | 1.75 | 0.83 | 1.06 | 0.99 | 1.00 | -0.21 | -0.38 | -0.51 | -0.89 | -0.25 | -0.41 | -0.40 | -0.41 |
|  | 6-31G** | 0.24 | 0.66 | 1.46 | 1.75 | 0.82 | 1.07 | 0.99 | 1.01 | -0.27 | -0.40 | -0.57 | -0.75 | -0.25 | -0.45 | -0.44 | -0.45 |
|  | 6-311G** | 0.17 | 0.91 | 1.48 | 1.73 | 0.80 | 1.11 | 1.03 | 1.04 | -0.06 | -0.32 | -0.54 | -0.74 | -0.25 | -0.46 | -0.45 | -0.46 |

Table S8. Charge of magnesium in MgCl_2_ and fluorine in HF, using different population methods, basis sets, and the MP2 method.

|  | | **Magnesium Charge in MgCl_2_ (ΔEN=1.85)** | | | | | | | | **Fluorine Charge in HF (ΔEN=1.88)** | | | | | | | |
| --- | --- | --- | --- | --- | --- | --- | --- | --- | --- | --- | --- | --- | --- | --- | --- | --- | --- |
|  |  | Q_LPA_ | Q_MPA_ | Q_NPA_ | Q_AIM_ | Q_HPA_ | Q_MK_ | Q_CHELP_ | Q_CHELPG_ | Q_LPA_ | Q_MPA_ | Q_NPA_ | Q_AIM_ | Q_HPA_ | Q_MK_ | Q_CHELP_ | Q_CHELPG_ |
| **MP2** | cc-pVDZ | 0.16 | 0.69 | 1.60 | 1.72 | 0.70 | 0.98 | 0.90 | 1.27 | -0.06 | -0.22 | -0.52 | -0.73 | -0.23 | -0.42 | -0.41 | -0.42 |
|  | cc-pVTZ | -0.30 | 0.77 | 1.66 | 1.74 | 0.71 | 0.99 | 0.90 | 1.27 | 0.25 | -0.32 | -0.53 | -0.74 | -0.22 | -0.42 | -0.40 | -0.42 |
|  | cc-pVQZ | -0.85 | 0.91 | 1.69 | 1.74 | 0.71 | 0.99 | 0.91 | 1.28 | 0.62 | -0.34 | -0.54 | -0.76 | -0.22 | -0.42 | -0.40 | -0.42 |
|  | cc-pV5Z | -1.14 | 0.90 | 1.69 | 1.74 | 0.71 | 1.00 | 0.91 | 1.28 | 0.85 | -0.34 | -0.53 | -0.76 | -0.21 | -0.42 | -0.40 | -0.42 |
|  | aug-cc-pVDZ | 0.22 | 0.98 | 1.70 | 1.74 | 0.69 | 0.99 | 0.90 | 0.69 | 0.08 | -0.27 | -0.56 | -0.72 | -0.21 | -0.42 | -0.40 | -0.41 |
|  | aug-cc-pVTZ | -0.62 | 0.76 | 1.70 | 1.74 | 0.70 | 0.99 | 0.91 | 0.70 | 0.66 | -0.34 | -0.55 | -0.75 | -0.21 | -0.42 | -0.40 | -0.41 |
|  | aug-cc-pVQZ | -1.42 | 1.07 | 1.70 | 1.74 | 0.71 | 1.00 | 0.91 | 0.71 | 1.13 | -0.39 | -0.55 | -0.76 | -0.21 | -0.42 | -0.40 | -0.41 |
|  | aug-cc-pV5Z | -2.20 | 1.24 | 1.70 | 1.74 | 0.71 | 1.00 | 0.91 | 0.71 | 1.35 | -0.50 | -0.53 | -0.76 | -0.21 | -0.42 | -0.40 | -0.41 |
|  | 6-21G** | 0.08 | 0.63 | 1.56 | 1.71 | 0.69 | 0.98 | 0.90 | 1.26 | -0.19 | -0.36 | -0.49 | -0.69 | -0.23 | -0.39 | -0.38 | -0.39 |
|  | 6-31G** | 0.18 | 0.58 | 1.56 | 1.71 | 0.69 | 0.99 | 0.91 | 1.27 | -0.26 | -0.37 | -0.54 | -0.72 | -0.23 | -0.43 | -0.42 | -0.43 |
|  | 6-311G** | 0.13 | 0.82 | 1.64 | 1.69 | 0.67 | 1.03 | 0.95 | 1.30 | -0.04 | -0.30 | -0.52 | -0.70 | -0.23 | -0.44 | -0.42 | -0.44 |

Table S9. Charge of bromine in BF_3_ and magnesium in MgO, using different population methods, basis sets, and quantum methods.

|  |  | **Bromine Charge in BF_3_ (ΔEN=1.94)** | | | | | | | | **Magnesium Charge in MgO (ΔEN=2.13)** | | | | | | | |
| --- | --- | --- | --- | --- | --- | --- | --- | --- | --- | --- | --- | --- | --- | --- | --- | --- | --- |
|  |  | Q_LPA_ | Q_MPA_ | Q_NPA_ | Q_AIM_ | Q_HPA_ | Q_MK_ | Q_CHELP_ | Q_CHELPG_ | Q_LPA_ | Q_MPA_ | Q_NPA_ | Q_AIM_ | Q_HPA_ | Q_MK_ | Q_CHELP_ | Q_CHELPG_ |
| **B3LYP** | cc-pVDZ | -0.59 | 0.22 | 1.46 | 2.39 | 0.43 | 0.84 | 0.96 | 0.89 | 0.27 | 0.55 | 0.95 | 1.17 | 0.58 | 0.81 | 0.79 | 0.78 |
|  | cc-pVTZ | -1.34 | 0.51 | 1.39 | 2.46 | 0.43 | 0.95 | 1.06 | 1.00 | 0.05 | 0.57 | 1.12 | 1.23 | 0.59 | 0.87 | 0.85 | 0.84 |
|  | cc-pVQZ | -2.11 | 0.83 | 1.42 | 2.48 | 0.43 | 0.97 | 1.08 | 1.03 | -0.17 | 0.58 | 1.20 | 1.24 | 0.59 | 0.90 | 0.89 | 0.88 |
|  | cc-pV5Z | -2.62 | 0.93 | 1.38 | 2.47 | 0.43 | 0.98 | 1.09 | 1.03 | -0.27 | 0.58 | 1.29 | 1.24 | 0.59 | 0.92 | 0.90 | 0.89 |
|  | aug-cc-pVDZ | -0.35 | 0.98 | 1.52 | 2.41 | 0.41 | 0.92 | 1.02 | 0.98 | 0.70 | 0.97 | 1.38 | 1.26 | 0.59 | 0.92 | 0.91 | 0.89 |
|  | aug-cc-pVTZ | -1.47 | 1.19 | 1.39 | 2.47 | 0.43 | 0.98 | 1.08 | 1.04 | 0.34 | 0.79 | 1.37 | 1.25 | 0.59 | 0.92 | 0.91 | 0.89 |
|  | aug-cc-pVQZ | -2.33 | 1.75 | 1.41 | 2.48 | 0.43 | 0.98 | 1.09 | 1.04 | 0.06 | 0.69 | 1.36 | 1.25 | 0.59 | 0.92 | 0.91 | 0.89 |
|  | aug-cc-pV5Z | -2.87 | 1.47 | 1.37 | 2.48 | 0.43 | 0.98 | 1.09 | 1.04 | -0.08 | 0.32 | 1.36 | 1.25 | 0.59 | 0.92 | 0.91 | 0.89 |
|  | 6-21G** | 0.09 | 0.81 | 1.31 | 2.48 | 0.44 | 0.71 | 0.83 | 0.77 | 0.12 | 0.40 | 0.77 | 1.13 | 0.53 | 0.72 | 0.70 | 0.69 |
|  | 6-31G** | 0.06 | 0.62 | 1.43 | 2.45 | 0.45 | 0.85 | 0.94 | 0.89 | 0.35 | 0.65 | 0.97 | 1.17 | 0.58 | 0.82 | 0.80 | 0.79 |
|  | 6-311G** | -0.51 | 0.48 | 1.37 | 2.36 | 0.43 | 0.94 | 1.05 | 0.99 | 0.28 | 0.63 | 1.01 | 1.17 | 0.58 | 0.84 | 0.83 | 0.82 |
| **PBE0** | cc-pVDZ | -0.58 | 0.25 | 1.46 | 2.39 | 0.42 | 0.84 | 0.95 | 0.89 | 0.29 | 0.58 | 0.98 | 1.20 | 0.59 | 0.84 | 0.83 | 0.81 |
|  | cc-pVTZ | -1.33 | 0.50 | 1.38 | 2.48 | 0.44 | 0.93 | 1.03 | 0.98 | 0.06 | 0.58 | 1.14 | 1.26 | 0.61 | 0.90 | 0.88 | 0.87 |
|  | cc-pVQZ | -2.11 | 0.83 | 1.41 | 2.50 | 0.43 | 0.95 | 1.05 | 1.01 | -0.16 | 0.60 | 1.22 | 1.28 | 0.61 | 0.93 | 0.92 | 0.90 |
|  | cc-pV5Z | -2.62 | 0.91 | 1.37 | 2.49 | 0.43 | 0.96 | 1.06 | 1.01 | -0.26 | 0.61 | 1.31 | 1.28 | 0.61 | 0.94 | 0.93 | 0.92 |
|  | aug-cc-pVDZ | -0.34 | 1.03 | 1.51 | 2.42 | 0.41 | 0.90 | 0.99 | 0.95 | 0.71 | 0.98 | 1.39 | 1.29 | 0.61 | 0.95 | 0.93 | 0.92 |
|  | aug-cc-pVTZ | -1.46 | 1.20 | 1.38 | 2.49 | 0.43 | 0.96 | 1.06 | 1.01 | 0.35 | 0.81 | 1.39 | 1.29 | 0.61 | 0.95 | 0.93 | 0.92 |
|  | aug-cc-pVQZ | -2.33 | 1.78 | 1.40 | 2.50 | 0.43 | 0.96 | 1.06 | 1.01 | 0.07 | 0.77 | 1.38 | 1.29 | 0.61 | 0.95 | 0.93 | 0.92 |
|  | aug-cc-pV5Z | -2.87 | 1.34 | 1.36 | 2.49 | 0.43 | 0.96 | 1.06 | 1.01 | -0.07 | 0.47 | 1.38 | 1.28 | 0.61 | 0.95 | 0.94 | 0.92 |
|  | 6-21G** | 0.11 | 0.85 | 1.32 | 2.49 | 0.44 | 0.73 | 0.83 | 0.78 | 0.14 | 0.42 | 0.80 | 1.16 | 0.55 | 0.75 | 0.74 | 0.72 |
|  | 6-31G** | 0.07 | 0.65 | 1.43 | 2.46 | 0.45 | 0.85 | 0.93 | 0.89 | 0.37 | 0.67 | 1.00 | 1.20 | 0.59 | 0.85 | 0.83 | 0.82 |
|  | 6-311G** | -0.50 | 0.47 | 1.37 | 2.37 | 0.43 | 0.92 | 1.02 | 0.97 | 0.30 | 0.64 | 1.04 | 1.20 | 0.60 | 0.87 | 0.86 | 0.85 |
| **HF** | cc-pVDZ | -0.38 | 0.57 | 1.67 | 2.52 | 0.54 | 1.09 | 1.16 | 1.13 | 0.43 | 0.74 | 1.12 | 1.30 | 0.69 | 0.98 | 0.96 | 0.96 |
|  | cc-pVTZ | -1.18 | 0.71 | 1.56 | 2.59 | 0.55 | 1.12 | 1.19 | 1.16 | 0.18 | 0.73 | 1.26 | 1.38 | 0.71 | 1.05 | 1.04 | 1.03 |
|  | cc-pVQZ | -2.01 | 1.01 | 1.59 | 2.61 | 0.55 | 1.13 | 1.20 | 1.18 | -0.06 | 0.79 | 1.34 | 1.41 | 0.72 | 1.09 | 1.08 | 1.06 |
|  | cc-pV5Z | -2.55 | 1.05 | 1.55 | 2.61 | 0.55 | 1.13 | 1.20 | 1.18 | -0.18 | 0.83 | 1.41 | 1.41 | 0.72 | 1.10 | 1.09 | 1.08 |
|  | aug-cc-pVDZ | -0.18 | 1.45 | 1.70 | 2.55 | 0.53 | 1.07 | 1.14 | 1.12 | 0.82 | 1.11 | 1.47 | 1.41 | 0.72 | 1.11 | 1.10 | 1.08 |
|  | aug-cc-pVTZ | -1.38 | 1.37 | 1.56 | 2.61 | 0.55 | 1.13 | 1.19 | 1.18 | 0.42 | 0.89 | 1.47 | 1.41 | 0.72 | 1.10 | 1.09 | 1.08 |
|  | aug-cc-pVQZ | -2.30 | 2.02 | 1.58 | 2.62 | 0.55 | 1.13 | 1.20 | 1.18 | 0.13 | 1.03 | 1.46 | 1.42 | 0.72 | 1.10 | 1.09 | 1.08 |
|  | aug-cc-pV5Z | -2.86 | 1.75 | 1.54 | 2.61 | 0.55 | 1.13 | 1.20 | 1.18 | -0.01 | 0.88 | 1.46 | 1.42 | 0.72 | 1.11 | 1.10 | 1.08 |
|  | 6-21G** | 0.33 | 1.20 | 1.58 | 2.61 | 0.56 | 0.98 | 1.03 | 1.03 | 0.26 | 0.53 | 0.91 | 1.23 | 0.64 | 0.85 | 0.83 | 0.82 |
|  | 6-31G** | 0.25 | 0.95 | 1.64 | 2.59 | 0.56 | 1.08 | 1.13 | 1.12 | 0.49 | 0.81 | 1.12 | 1.30 | 0.69 | 0.98 | 0.96 | 0.96 |
|  | 6-311G** | -0.32 | 0.71 | 1.56 | 2.51 | 0.55 | 1.13 | 1.20 | 1.18 | 0.42 | 0.79 | 1.15 | 1.30 | 0.69 | 1.00 | 0.98 | 0.98 |

Table S10. Charge of bromine in BF_3_ and magnesium in MgO, using different population methods, basis sets, and the MP2 method.

|  | | **Bromine Charge in BF_3_ (ΔEN=1.94)** | | | | | | | | **Magnesium Charge in MgO (ΔEN=2.13)** | | | | | | | |
| --- | --- | --- | --- | --- | --- | --- | --- | --- | --- | --- | --- | --- | --- | --- | --- | --- | --- |
|  |  | Q_LPA_ | Q_MPA_ | Q_NPA_ | Q_AIM_ | Q_HPA_ | Q_MK_ | Q_CHELP_ | Q_CHELPG_ | Q_LPA_ | Q_MPA_ | Q_NPA_ | Q_AIM_ | Q_HPA_ | Q_MK_ | Q_CHELP_ | Q_CHELPG_ |
| **MP2** | cc-pVDZ | -0.54 | 0.34 | 1.46 | 2.43 | 0.42 | 0.94 | 1.02 | 1.05 | 0.21 | 0.50 | 1.08 | 1.24 | 0.53 | 0.84 | 0.82 | 1.18 |
|  | cc-pVTZ | -1.31 | 0.49 | 1.40 | 2.52 | 0.42 | 0.99 | 1.06 | 1.08 | -0.01 | 0.46 | 1.20 | 1.29 | 0.54 | 0.87 | 0.85 | 1.18 |
|  | cc-pVQZ | -2.10 | 0.79 | 1.44 | 2.54 | 0.43 | 0.98 | 1.08 | 1.08 | -0.26 | 0.54 | 1.29 | 1.29 | 0.55 | 0.90 | 0.88 | 1.18 |
|  | cc-pV5Z | -2.60 | 0.86 | 1.41 | 2.54 | 0.43 | 0.98 | 1.08 | 1.08 | -0.28 | 0.65 | 1.38 | 1.29 | 0.55 | 0.91 | 0.89 | 1.17 |
|  | aug-cc-pVDZ | -0.31 | 1.17 | 1.55 | 2.47 | 0.40 | 0.91 | 1.04 | 1.00 | 0.68 | 0.96 | 1.46 | 1.30 | 0.54 | 0.92 | 0.91 | 1.12 |
|  | aug-cc-pVTZ | -1.46 | 1.08 | 1.42 | 2.54 | 0.42 | 0.96 | 1.08 | 1.05 | 0.35 | 0.69 | 1.47 | 1.32 | 0.55 | 0.93 | 0.92 | 1.13 |
|  | aug-cc-pVQZ | -2.33 | 1.73 | 1.44 | 2.55 | 0.43 | 0.98 | 1.08 | 1.08 | 0.06 | 0.85 | 1.48 | 1.32 | 0.55 | 0.93 | 0.92 | 1.15 |
|  | aug-cc-pV5Z | -2.87 | 1.49 | 1.41 | 2.54 | 0.43 | 0.98 | 1.08 | 1.08 | 0.09 | 0.73 | 1.47 | 1.31 | 0.55 | 0.92 | 0.91 | 1.16 |
|  | 6-21G** | -0.47 | 0.96 | 1.35 | 2.54 | 0.44 | 0.80 | 0.89 | 0.90 | 0.09 | 0.36 | 0.94 | 1.28 | 0.52 | 0.81 | 0.80 | 1.12 |
|  | 6-31G** | 0.10 | 0.72 | 1.44 | 2.51 | 0.43 | 0.91 | 0.99 | 1.00 | 0.30 | 0.60 | 1.09 | 1.24 | 0.54 | 0.85 | 0.83 | 1.13 |
|  | 6-311G** | 0.16 | 0.52 | 1.36 | 2.42 | 0.42 | 0.97 | 1.07 | 1.05 | 0.20 | 0.57 | 1.12 | 1.22 | 0.53 | 0.84 | 0.82 | 1.12 |

Table S11. Charge of sodium in NaCl and fluorine in LiF, using different population methods, basis sets, and quantum methods.

| ­­ |  | **Sodium Charge in NaCl (ΔEN=2.23)** | | | | | | | | **Fluorine Charge in LiF (ΔEN=3.00)** | | | | | | | |
| --- | --- | --- | --- | --- | --- | --- | --- | --- | --- | --- | --- | --- | --- | --- | --- | --- | --- |
|  |  | Q_LPA_ | Q_MPA_ | Q_NPA_ | Q_AIM_ | Q_HPA_ | Q_MK_ | Q_CHELP_ | Q_CHELPG_ | Q_LPA_ | Q_MPA_ | Q_NPA_ | Q_AIM_ | Q_HPA_ | Q_MK_ | Q_CHELP_ | Q_CHELPG_ |
| **B3LYP** | cc-pVDZ | 0.18 | 0.54 | 0.84 | 0.88 | 0.59 | 0.75 | 0.79 | 0.75 | -0.23 | -0.47 | -0.81 | * | -0.57 | -0.80 | -0.80 | -0.81 |
|  | cc-pVTZ | -0.13 | 0.52 | 0.88 | 0.89 | 0.59 | 0.76 | 0.79 | 0.75 | 0.11 | -0.44 | -0.88 | -0.92 | -0.57 | -0.81 | -0.81 | -0.81 |
|  | cc-pVQZ | -0.44 | 0.54 | 0.90 | 0.88 | 0.59 | 0.76 | 0.80 | 0.75 | 0.49 | -0.48 | -0.90 | -0.92 | -0.57 | -0.81 | -0.81 | -0.82 |
|  | cc-pV5Z | -0.77 | 0.63 | 0.90 | 0.88 | 0.58 | 0.77 | 0.80 | 0.75 | 0.73 | -0.64 | -0.91 | -0.92 | -0.57 | -0.82 | -0.82 | -0.82 |
|  | aug-cc-pVDZ | 0.38 | 0.76 | 0.91 | 0.89 | 0.59 | 0.78 | 0.81 | 0.76 | -0.49 | -0.63 | -0.93 | -0.94 | -0.57 | -0.83 | -0.83 | -0.83 |
|  | aug-cc-pVTZ | -0.07 | 0.68 | 0.91 | 0.89 | 0.58 | 0.77 | 0.80 | 0.76 | -0.06 | -0.64 | -0.91 | -0.92 | -0.57 | -0.82 | -0.82 | -0.82 |
|  | aug-cc-pVQZ | -0.47 | 0.52 | 0.91 | 0.88 | 0.58 | 0.77 | 0.80 | 0.75 | 0.34 | -0.60 | -0.91 | -0.92 | -0.57 | -0.82 | -0.82 | -0.82 |
|  | aug-cc-pV5Z | -0.88 | 0.53 | 0.91 | 0.88 | 0.58 | 0.77 | 0.80 | 0.75 | 0.61 | -0.89 | -0.91 | -0.92 | -0.57 | -0.82 | -0.82 | -0.82 |
|  | 6-21G** | 0.28 | 0.57 | 0.83 | 0.87 | 0.59 | 0.76 | 0.80 | 0.75 | -0.30 | -0.41 | -0.64 | -0.81 | -0.53 | -0.64 | -0.63 | -0.65 |
|  | 6-31G** | 0.31 | 0.56 | 0.85 | 0.88 | 0.60 | 0.77 | 0.80 | 0.76 | -0.30 | -0.53 | -0.80 | * | -0.58 | -0.76 | -0.75 | -0.77 |
|  | 6-311G** | 0.36 | 0.68 | 0.90 | 0.88 | 0.58 | 0.79 | 0.83 | 0.78 | -0.18 | -0.56 | -0.84 | * | -0.58 | -0.77 | -0.77 | -0.78 |
| **PBE0** | cc-pVDZ | 0.20 | 0.57 | 0.86 | 0.90 | 0.61 | 0.77 | 0.81 | 0.77 | -0.25 | -0.50 | -0.82 | * | -0.59 | -0.81 | -0.81 | -0.82 |
|  | cc-pVTZ | -0.12 | 0.53 | 0.89 | 0.90 | 0.60 | 0.77 | 0.80 | 0.76 | 0.09 | -0.46 | -0.88 | -0.92 | -0.58 | -0.81 | -0.81 | -0.82 |
|  | cc-pVQZ | -0.43 | 0.55 | 0.91 | 0.89 | 0.60 | 0.77 | 0.81 | 0.76 | 0.48 | -0.50 | -0.90 | -0.92 | -0.58 | -0.82 | -0.82 | -0.82 |
|  | cc-pV5Z | -0.76 | 0.63 | 0.91 | 0.89 | 0.60 | 0.78 | 0.81 | 0.76 | 0.72 | -0.65 | -0.91 | -0.92 | -0.58 | -0.82 | -0.82 | -0.83 |
|  | aug-cc-pVDZ | 0.39 | 0.79 | 0.92 | 0.90 | 0.60 | 0.78 | 0.82 | 0.77 | -0.50 | -0.65 | -0.93 | -0.94 | -0.59 | -0.83 | -0.83 | -0.84 |
|  | aug-cc-pVTZ | -0.06 | 0.72 | 0.92 | 0.90 | 0.60 | 0.78 | 0.81 | 0.77 | -0.07 | -0.66 | -0.92 | -0.93 | -0.58 | -0.82 | -0.83 | -0.83 |
|  | aug-cc-pVQZ | -0.46 | 0.55 | 0.92 | 0.89 | 0.60 | 0.78 | 0.81 | 0.76 | 0.33 | -0.63 | -0.92 | -0.93 | -0.58 | -0.82 | -0.83 | -0.83 |
|  | aug-cc-pV5Z | -0.88 | 0.56 | 0.92 | 0.89 | 0.60 | 0.78 | 0.81 | 0.76 | 0.61 | -0.91 | -0.91 | -0.92 | -0.58 | -0.82 | -0.83 | -0.83 |
|  | 6-21G** | 0.31 | 0.60 | 0.85 | 0.89 | 0.61 | 0.78 | 0.82 | 0.77 | -0.33 | -0.44 | -0.67 | -0.83 | -0.55 | -0.66 | -0.66 | -0.67 |
|  | 6-31G** | 0.33 | 0.59 | 0.87 | 0.89 | 0.61 | 0.78 | 0.82 | 0.78 | -0.32 | -0.55 | -0.82 | * | -0.59 | -0.77 | -0.77 | -0.78 |
|  | 6-311G** | 0.37 | 0.69 | 0.91 | 0.89 | 0.60 | 0.81 | 0.84 | 0.79 | -0.20 | -0.58 | -0.85 | * | -0.59 | -0.78 | -0.78 | -0.79 |
| **HF** | cc-pVDZ | 0.24 | 0.64 | 0.90 | 0.92 | 0.64 | 0.82 | 0.85 | 0.81 | -0.34 | -0.62 | -0.89 | * | -0.64 | -0.86 | -0.86 | -0.87 |
|  | cc-pVTZ | -0.09 | 0.59 | 0.92 | 0.92 | 0.64 | 0.81 | 0.84 | 0.80 | 0.02 | -0.57 | -0.92 | * | -0.64 | -0.86 | -0.86 | -0.86 |
|  | cc-pVQZ | -0.42 | 0.64 | 0.93 | 0.92 | 0.63 | 0.81 | 0.84 | 0.80 | 0.42 | -0.61 | -0.93 | -0.94 | -0.63 | -0.86 | -0.86 | -0.86 |
|  | cc-pV5Z | -0.76 | 0.64 | 0.93 | 0.92 | 0.63 | 0.81 | 0.84 | 0.80 | 0.69 | -0.74 | -0.94 | -0.94 | -0.63 | -0.86 | -0.86 | -0.86 |
|  | aug-cc-pVDZ | 0.40 | 0.82 | 0.94 | 0.92 | 0.63 | 0.82 | 0.85 | 0.81 | -0.55 | -0.71 | -0.95 | -0.95 | -0.64 | -0.87 | -0.87 | -0.87 |
|  | aug-cc-pVTZ | -0.06 | 0.76 | 0.94 | 0.92 | 0.63 | 0.82 | 0.84 | 0.81 | -0.11 | -0.77 | -0.94 | -0.94 | -0.63 | -0.86 | -0.86 | -0.86 |
|  | aug-cc-pVQZ | -0.47 | 0.75 | 0.94 | 0.92 | 0.63 | 0.82 | 0.84 | 0.80 | 0.30 | -0.82 | -0.94 | -0.94 | -0.63 | -0.86 | -0.86 | -0.86 |
|  | aug-cc-pV5Z | -0.89 | 0.74 | 0.94 | 0.92 | 0.63 | 0.81 | 0.84 | 0.80 | 0.60 | -1.01 | -0.94 | -0.94 | -0.63 | -0.86 | -0.86 | -0.86 |
|  | 6-21G** | 0.36 | 0.68 | 0.89 | 0.91 | 0.65 | 0.83 | 0.86 | 0.82 | -0.46 | -0.59 | -0.78 | * | -0.63 | -0.75 | -0.75 | -0.76 |
|  | 6-31G** | 0.37 | 0.66 | 0.91 | 0.92 | 0.64 | 0.83 | 0.86 | 0.82 | -0.41 | -0.66 | -0.88 | * | -0.65 | -0.83 | -0.83 | -0.84 |
|  | 6-311G** | 0.40 | 0.75 | 0.94 | 0.92 | 0.63 | 0.85 | 0.87 | 0.84 | -0.28 | -0.69 | -0.91 | * | -0.64 | -0.84 | -0.84 | -0.85 |

*Program unable to run this analysis method with the indicated method/basis set combination due to large ΔEN.

Table S12. Charge of sodium in NaCl and fluorine in LiF, using different population methods, basis sets, and the MP2 method.

|  | | **Sodium Charge in NaCl (ΔEN=2.23)** | | | | | | | | **Fluorine Charge in LiF (ΔEN=3.00)** | | | | | | | |
| --- | --- | --- | --- | --- | --- | --- | --- | --- | --- | --- | --- | --- | --- | --- | --- | --- | --- |
|  |  | Q_LPA_ | Q_MPA_ | Q_NPA_ | Q_AIM_ | Q_HPA_ | Q_MK_ | Q_CHELP_ | Q_CHELPG_ | Q_LPA_ | Q_MPA_ | Q_NPA_ | Q_AIM_ | Q_HPA_ | Q_MK_ | Q_CHELP_ | Q_CHELPG_ |
| **MP2** | cc-pVDZ | 0.22 | 0.59 | 0.89 | 0.91 | 0.60 | 0.80 | 0.84 | 0.83 | -0.25 | -0.50 | -0.86 | * | -0.58 | -0.82 | -0.82 | -0.87 |
|  | cc-pVTZ | -0.11 | 0.54 | 0.93 | 0.92 | 0.60 | 0.80 | 0.83 | 0.83 | 0.09 | -0.47 | -0.92 | -0.92 | -0.58 | -0.82 | -0.82 | -0.86 |
|  | cc-pVQZ | -0.46 | 0.57 | 0.94 | 0.92 | 0.60 | 0.80 | 0.83 | 0.83 | 0.47 | -0.52 | -0.94 | -0.93 | -0.58 | -0.83 | -0.83 | -0.86 |
|  | cc-pV5Z | -0.73 | 0.64 | 0.95 | 0.91 | 0.60 | 0.80 | 0.83 | 0.83 | 0.72 | -0.67 | -0.95 | -0.93 | -0.58 | -0.83 | -0.83 | -0.86 |
|  | aug-cc-pVDZ | 0.40 | 0.81 | 0.96 | 0.91 | 0.60 | 0.81 | 0.85 | 0.84 | -0.50 | -0.65 | -0.97 | -0.94 | -0.58 | -0.84 | -0.84 | -0.85 |
|  | aug-cc-pVTZ | -0.06 | 0.70 | 0.96 | 0.92 | 0.60 | 0.81 | 0.84 | 0.84 | -0.07 | -0.71 | -0.96 | -0.93 | -0.58 | -0.84 | -0.84 | -0.85 |
|  | aug-cc-pVQZ | -0.50 | 0.64 | 0.95 | 0.92 | 0.60 | 0.81 | 0.84 | 0.83 | 0.33 | -0.75 | -0.95 | -0.93 | -0.58 | -0.84 | -0.84 | -0.86 |
|  | aug-cc-pV5Z | -0.86 | 0.70 | 0.95 | 0.91 | 0.60 | 0.80 | 0.83 | 0.83 | 0.61 | -0.93 | -0.95 | -0.93 | -0.58 | -0.84 | -0.84 | -0.86 |
|  | 6-21G** | 0.32 | 0.61 | 0.88 | 0.90 | 0.60 | 0.80 | 0.83 | 0.83 | -0.34 | -0.46 | -0.74 | -0.84 | -0.55 | -0.67 | -0.67 | -0.74 |
|  | 6-31G** | 0.33 | 0.60 | 0.90 | 0.90 | 0.60 | 0.80 | 0.83 | 0.83 | -0.33 | -0.56 | -0.86 | * | -0.59 | -0.78 | -0.78 | -0.82 |
|  | 6-311G** | 0.37 | 0.70 | 0.94 | 0.90 | 0.59 | 0.82 | 0.85 | 0.85 | -0.20 | -0.59 | -0.89 | * | -0.59 | -0.79 | -0.79 | -0.83 |

Table S13. Charge of vanadium in VO using different population methods, basis sets, and quantum methods.

| ­ | | **Vanadium Charge in VO** | | | | | | | |
| --- | --- | --- | --- | --- | --- | --- | --- | --- | --- |
|  |  | Q_LPA_ | Q_MPA_ | Q_NPA_ | Q_AIM_ | Q_HPA_ | Q_MK_ | Q_CHELP_ | Q_CHELPG_ |
| **B3LYP** | cc-pVDZ | -0.15 | 0.28 | 1.96 | 0.83 | 0.35 | 0.85 | 0.54 | 0.93 |
|  | cc-pVTZ | -0.09 | 0.41 | 1.96 | 0.85 | 0.36 | 0.85 | 0.55 | 0.93 |
|  | cc-pVQZ | -0.18 | 0.47 | 1.96 | 0.86 | 0.36 | 0.85 | 0.55 | 0.93 |
|  | cc-pV5Z | -0.23 | 0.43 | 1.96 | 0.86 | 0.36 | 0.86 | 0.55 | 0.93 |
|  | aug-cc-pVDZ | 0.16 | 0.48 | 1.97 | 0.86 | 0.36 | 0.86 | 0.55 | 0.93 |
|  | aug-cc-pVTZ | 0.14 | 0.54 | 1.96 | 0.86 | 0.36 | 0.86 | 0.55 | 0.93 |
|  | aug-cc-pVQZ | 0.10 | 0.48 | 1.97 | 0.87 | 0.36 | 0.86 | 0.55 | 0.93 |
|  | aug-cc-pV5Z | 0.12 | 0.36 | 1.97 | 0.86 | 0.36 | 0.86 | 0.55 | 0.93 |
| **PBE0** | cc-pVDZ | -0.08 | 0.36 | 2.07 | 0.92 | 0.41 | 0.86 | 0.55 | 0.93 |
|  | cc-pVTZ | -0.02 | 0.47 | 2.07 | 0.94 | 0.42 | 0.86 | 0.55 | 0.93 |
|  | cc-pVQZ | -0.13 | 0.53 | 2.08 | 0.95 | 0.42 | 0.86 | 0.55 | 0.94 |
|  | cc-pV5Z | -0.17 | 0.49 | 2.08 | 0.95 | 0.42 | 0.86 | 0.56 | 0.94 |
|  | aug-cc-pVDZ | 0.23 | 0.55 | 2.09 | 0.94 | 0.42 | 0.86 | 0.56 | 0.94 |
|  | aug-cc-pVTZ | 0.19 | 0.57 | 2.08 | 0.95 | 0.42 | 0.86 | 0.56 | 0.94 |
|  | aug-cc-pVQZ | 0.14 | 0.56 | 2.08 | 0.96 | 0.42 | 0.86 | 0.56 | 0.94 |
|  | aug-cc-pV5Z | 0.16 | 0.43 | 2.08 | 0.96 | 0.42 | 0.86 | 0.56 | 0.94 |
| **HF** | cc-pVDZ | -0.05 | 0.42 | 2.26 | 0.89 | 0.40 | 0.83 | 0.42 | 0.90 |
|  | cc-pVTZ | -0.01 | 0.54 | 2.26 | 0.91 | 0.41 | 0.83 | 0.42 | 0.91 |
|  | cc-pVQZ | -0.13 | 0.58 | 2.26 | 0.92 | 0.41 | 0.83 | 0.42 | 0.91 |
|  | cc-pV5Z | -0.19 | 0.55 | 2.26 | 0.92 | 0.41 | 0.83 | 0.42 | 0.91 |
|  | aug-cc-pVDZ | 0.22 | 0.61 | 2.27 | 0.92 | 0.41 | 0.84 | 0.43 | 0.92 |
|  | aug-cc-pVTZ | 0.15 | 0.60 | 2.26 | 0.92 | 0.41 | 0.84 | 0.42 | 0.91 |
|  | aug-cc-pVQZ | 0.09 | 0.61 | 2.27 | 0.92 | 0.41 | 0.83 | 0.42 | 0.91 |
|  | aug-cc-pV5Z | 0.11 | 0.50 | 2.27 | 0.92 | 0.41 | 0.83 | 0.42 | 0.91 |

Table S14. Charge of vanadium in VO using different population methods, basis sets, and the MP2 method.

|  | | **Vanadium Charge in VO** | | | | | | | |
| --- | --- | --- | --- | --- | --- | --- | --- | --- | --- |
|  |  | Q_LPA_ | Q_MPA_ | Q_NPA_ | Q_AIM_ | Q_HPA_ | Q_MK_ | Q_CHELP_ | Q_CHELPG_ |
| **MP2** | cc-pVDZ | -0.04 | 0.34 | 1.98 | 1.17 | 0.54 | 1.19 | 0.99 | 1.25 |
|  | cc-pVTZ | 0.09 | 0.49 | 1.96 | 1.29 | 0.59 | 1.24 | 1.06 | 1.31 |
|  | cc-pVQZ | 0.02 | 0.60 | 1.97 | 1.32 | 0.60 | 1.25 | 1.07 | 1.32 |
|  | cc-pV5Z | 0.00 | 0.58 | 1.97 | 1.33 | 0.60 | 1.26 | 1.08 | 1.33 |
|  | aug-cc-pVDZ | 0.40 | 0.60 | 1.97 | 1.32 | 0.59 | 1.29 | 1.12 | 1.35 |
|  | aug-cc-pVTZ | 0.38 | 0.63 | 1.97 | 1.33 | 0.60 | 1.27 | 1.09 | 1.33 |
|  | aug-cc-pVQZ | 0.34 | 0.67 | 1.98 | 1.33 | 0.60 | 1.27 | 1.09 | 1.33 |
|  | aug-cc-pV5Z | 0.36 | 0.54 | 1.98 | 1.34 | 0.60 | 1.26 | 1.09 | 1.33 |

Table S15. Charge of lawrencium in LrF using different population methods, basis sets, and quantum methods.

| ­ | | **Lawrencium Charge in LrF** | | | | |
| --- | --- | --- | --- | --- | --- | --- |
|  |  | Q_LPA_ | Q_MPA_ | Q_NPA_ | Q_AIM_ | Q_HPA_ |
| **B3LYP** | cc-pVDZ-DK3 | 0.22 | 0.36 | 0.74 | 0.73 | 0.36 |
|  | cc-pVTZ-DK3 | 0.04 | 0.43 | 0.79 | 0.76 | 0.36 |
|  | cc-pVQZ-DK3 | -0.04 | 0.47 | 0.80 | 0.77 | 0.36 |
|  | cc-pwCVDZ-DK3 | 0.21 | 0.36 | 0.74 | 0.73 | 0.36 |
|  | cc-pwCVTZ-DK3 | 0.04 | 0.44 | 0.79 | 0.76 | 0.36 |
|  | cc-pwCVQZ-DK3 | -0.04 | 0.47 | 0.80 | 0.77 | 0.36 |
| **PBE0** | cc-pVDZ-DK3 | 0.23 | 0.38 | 0.75 | 0.73 | 0.36 |
|  | cc-pVTZ-DK3 | 0.04 | 0.43 | 0.80 | 0.77 | 0.37 |
|  | cc-pVQZ-DK3 | -0.03 | 0.46 | 0.80 | 0.78 | 0.37 |
|  | cc-pwCVDZ-DK3 | 0.23 | 0.38 | 0.75 | 0.73 | 0.36 |
|  | cc-pwCVTZ-DK3 | 0.05 | 0.44 | 0.80 | 0.76 | 0.37 |
|  | cc-pwCVQZ-DK3 | -0.04 | 0.46 | 0.80 | 0.78 | 0.37 |
| **HF** | cc-pVDZ-DK3 | 0.34 | 0.54 | 0.85 | 0.83 | 0.45 |
|  | cc-pVTZ-DK3 | 0.13 | 0.56 | 0.87 | 0.85 | 0.45 |
|  | cc-pVQZ-DK3 | 0.03 | 0.57 | 0.88 | 0.86 | 0.45 |
|  | cc-pwCVDZ-DK3 | 0.34 | 0.54 | 0.85 | 0.83 | 0.45 |
|  | cc-pwCVTZ-DK3 | 0.14 | 0.56 | 0.87 | 0.85 | 0.45 |
|  | cc-pwCVQZ-DK3 | 0.03 | 0.57 | 0.88 | 0.86 | 0.45 |

Table S16. Charge of lawrencium in LrF using different population methods, basis sets, and the MP2.

|  | | **Lawrencium Charge in LrF** | | | | |
| --- | --- | --- | --- | --- | --- | --- |
|  |  | Q_LPA_ | Q_MPA_ | Q_NPA_ | Q_AIM_ | Q_HPA_ |
| **MP2** | cc-pVDZ-DK3 | 0 | 0.40 | 0.75 | 0.76 | 0.37 |
|  | cc-pVTZ-DK3 | 0.05 | 0.44 | 0.79 | 0.79 | 0.37 |
|  | cc-pVQZ-DK3 | -0.03 | 0.47 | 0.79 | 0.80 | 0.37 |
|  | cc-pwCVDZ-DK3 | 0.23 | 0.39 | 0.73 | 0.75 | 0.36 |
|  | cc-pwCVTZ-DK3 | 0.05 | 0.43 | 0.77 | 0.78 | 0.37 |
|  | cc-pwCVQZ-DK3 | -0.04 | 0.46 | 0.78 | 0.79 | 0.37 |

Figure S1. Atomic charge on carbon in CH_4_, using Lӧwdin population analysis with the a) Dunning and b) Pople basis sets.


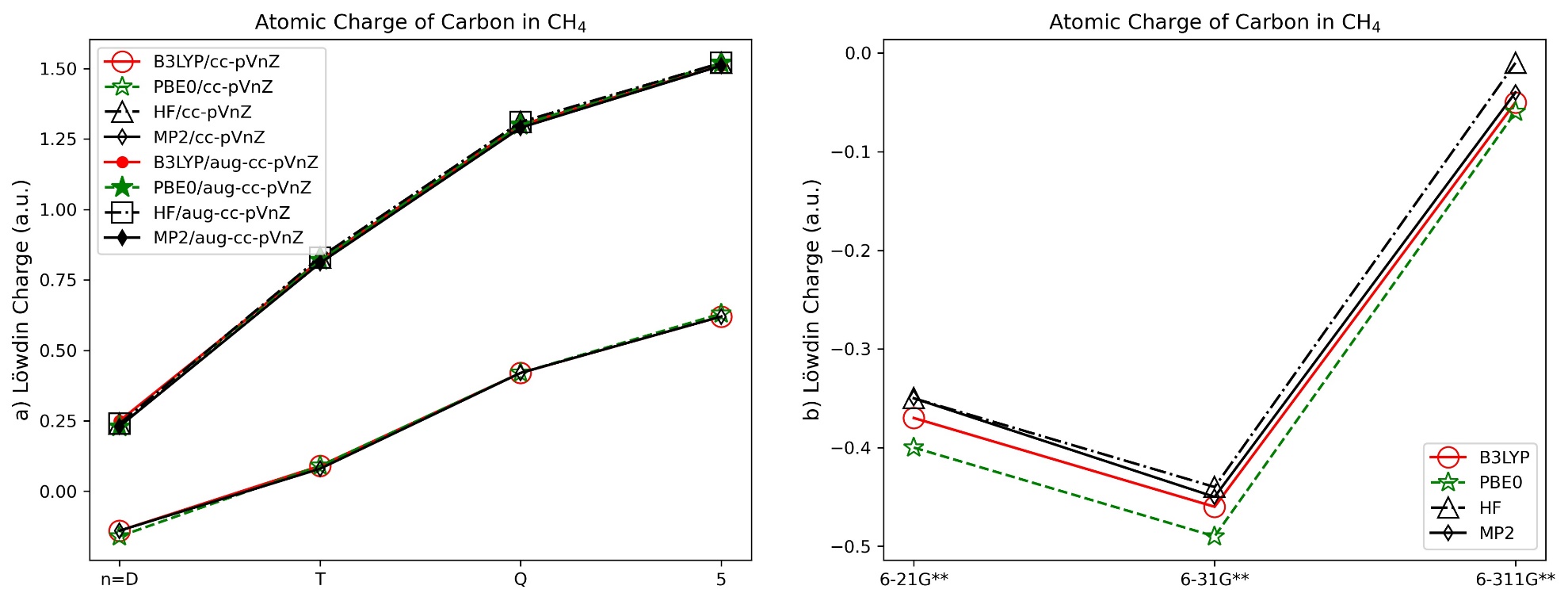

Supplement: Supplementary file 1 [file DataSheet1.docx]
